# Pharmacological Microglial Inhibition Remodels the Scar Microenvironment to Support Reticulospinal Circuit Reconstruction After Spinal Cord Injury

**DOI:** 10.1002/advs.202503966

**Published:** 2025-10-17

**Authors:** Run Li, Hongyuan Xing, Yifan Shen, Meng Chen, Bowen Lyu, Xiaofeng Yang, Li Sun, Chao Jiang, Jianyu Lv, Xin Ding, Zhongyang Gao, Yue Wang

**Affiliations:** ^1^ Department of Orthopedic Surgery The First Affiliated Hospital Zhejiang University School of Medicine Hangzhou 310003 China; ^2^ M.S.E Johns Hopkins University 3400 North Charles Street Hopkins MD 21218 USA; ^3^ Soochow Key Laboratory of Prevention and Treatment of Child Brain injury Children's Hospital of Soochow University Suzhou 215025 China; ^4^ Department of Orthopedics Taizhou Hospital of Zhejiang Province Affiliated to Wenzhou Medical University Taizhou 317000 China; ^5^ Department of Gastroenterology The Second Affiliated Hospital Zhejiang Chinese Medical University Hangzhou 310005 China; ^6^ Department of Neonatology Children's Hospital of Soochow University Suzhou 215000 China

**Keywords:** Î^2^2‐adrenergic receptor, microglial inhibition, motor function recovery, reticulospinal tract, spinal cord injury

## Abstract

Due to an inhibitory scar microenvironment that prevents neural circuit reconstruction, spinal cord injury (SCI) often leads to persistent neurological dysfunction. Although neonatal murine models demonstrate that microglial inhibition enables scar remodeling to support neuroregeneration and functional recovery, effective pharmacological suppression of microglial activation in adult SCI remain elusive. Here, this work demonstrates that early β2‐adrenergic receptor agonist treatment drives microglial transition to a homeostatic phenotype within the post‐SCI scar. This intervention reduces inhibitory extracellular matrix deposition and transforms the inhibitory microenvironments into permissive substrates for axonal regrowth. Anatomical analyses reveal regeneration of the reticulospinal tract, which establishes synaptic connectivity with thoracolumbar circuits to mediate motor recovery in a complete SCI. These findings elucidate the therapeutic potential and neural circuit mechanisms underlying pharmacological microglial modulation for SCI repair, establishing a glial‐neural circuit reparative paradigm.

## Graphical abstract

This study establishes pharmacological microglial inhibition as a therapeutic strategy, demonstrating its capacity to remodel the lesion microenvironment through significant reduction of extracellular matrix deposition. This permissive environmental transformation facilitates robust regeneration of the reticulospinal tract, and reconstructs functional synapses with local spinal circuits, and ultimately restores motor function.

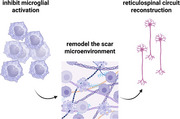

## 1 Introduction

Spinal cord injury (SCI) is a devastating neurological disorder that results in permanent loss of sensory and motor functions^[^ ^1^, ^2^ ^]^ The pathological cascade following SCI features progressive scar formation at the lesion site,^[^ ^3^, ^4^ ^]^ comprising aberrantly proliferating astrocytes, pericytes, fibroblasts, activated microglia, and multiple inhibitory factors of neural regeneration.^[^ ^5^ ^]^ This inhibitory scar environment serves as a principal contributor to secondary neurodegeneration, suppresses axonal regeneration and ultimately leads to failure of functional recovery.^[^ ^6^ ^]^ Consequently, remodeling the scar microenvironment has emerged as a promising therapeutic strategy to promote central nervous system (CNS) axon regeneration and functional recovery.^[^ ^5^, ^6^, ^7^, ^8^ ^]^

Recent studies indicate that targeting microglial activation is a promising therapeutic approach to remodel the scar environment after SCI.^[^ ^9^, ^10^ ^]^ Upon activation, microglia secrete various pro‐inflammatory mediators, such as cytokines, chemokines, and reactive oxygen,^[^ ^11^ ^]^ to establish an inflammatory microenvironment at the SCI site. Moreover, activated microglia induces secondary activation of neighboring astroglia cells. Intriguingly, neonatal murine models exhibit intrinsic microglial phenotypic plasticity, whereby activated microglia spontaneously return to a resting state.^[^ ^12^, ^13^, ^14^, ^15^ ^]^ This phenotypic transition effectively prevents scar formation and enables remarkable axonal regeneration across complete SCI lesions.^[^ ^12^, ^16^ ^]^ Interventions targeting microglial activation have shown therapeutic effects in treating Parkinson's disease and Alzheimer's disease,^[^ ^17^, ^18^ ^]^ supporting the concept that modulating microglial state can improve CNS outcomes. By extension, and together with the neonatal plasticity noted above, inhibiting microglia after SCI may help remodel the scar environment, promote reconstruction of neural circuits and ultimately improve functional recovery. To date, effective pharmacological methods to effectively inhibit microglial activation after SCI remain lacking. Moreover, conventional microglial depletion strategies paradoxically exacerbate scar formation and neuroinflammation at the SCI site,^[^ ^19^, ^20^ ^]^ likely reflecting the dual roles of microglia. Emerging evidence highlights their neuroprotective functions including wound weaving and compaction of the injury site,^[^ ^21^ ^]^ necessitating refined pharmacological strategies that selectively temper microglial activation.

As a key member of the G protein‐coupled receptor (GPCR) superfamily, β2‐adrenergic receptor (Adrb2) is a canonical heptahelical GPCR that couples with Gs proteins to activate adenylate cyclase and elevate intracellular cyclic adenosine monophosphate levels to trigger downstream signaling pathways.^[^ ^22^, ^23^ ^]^ Adrb2 mediates physiological effects of catecholamines and plays a central role in cardiovascular, respiratory, metabolic, and immunomodulatory functions.^[^ ^24^, ^25^ ^]^ In neural tissues, Adrb2 is predominantly expressed in microglia. Under physiological conditions, neural signaling modulates microglial functions via Adrb2.^[^ ^26^ ^]^ Neural synapses release norepinephrine to activate Adrb2 on microglia and thereby, reduce microglial environmental surveillance and downregulate inflammatory responses.^[^ ^26^, ^27^ ^]^ Given the regulatory roles in microglia, Adrb2 agonists, such as clenbuterol, have been demonstrated to exert neuroprotective effects and promote tissue repair.^[^ ^28^, ^29^, ^30^ ^]^ As such, it is possible that an Adrb2 agonist inhibits aberrant microglial activities within the SCI microenvironment and act as a therapeutic agent to improve spinal cord repairing.

Despite significant advances in remodeling scar environment and promoting neural regeneration,^[^ ^7^, ^31^, ^32^, ^33^ ^]^ it remains unknown which descending tracts are critical for motor recovery with these strategies.^[^ ^34^ ^]^ In murine models, coordinated motor control emerges from integrated neural networks involving multiple descending tracts, particularly the corticospinal (CST) and reticulospinal (ReST) pathways, which synergistically regulate locomotor patterning, postural adjustment, and voluntary movement execution.^[^ ^35^, ^36^ ^]^ While damage to these critical pathways undoubtedly results in impaired motor function,^[^ ^34^, ^37^, ^38^ ^]^ electrophysiological neuromodulation or pro‐regeneration strategies have demonstrated enhanced circuit plasticity of these pathways, which mediating the functional restoration after SCI.^[^ ^38^, ^39^ ^]^ Therefore, elucidating tract‐specific regeneration patterns is essential for scar‐targeted therapies in SCI.

Using transgenic animals and in vivo two‐photon imaging, we demonstrated that early, but not delayed, Adrb2 agonist treatment suppresses microglial activation within the post‐SCI scar microenvironment and promotes a transition to a resting state. This, in turn, attenuates inhibitory fibrotic scar formation and remodels the scar microenvironment into a permissive substrate for central axonal regeneration, ultimately improving motor recovery in a complete SCI model. Structurally, the ReST, rather than CST, axons successfully cross the injury site and establish functional synaptic connections with caudal spinal circuits. Together, these findings demonstrate that Adrb2‐agonist‐mediated microglial modulation mitigates inhibitory scarring and promotes neural‐circuit reconstruction, informing the design of effective therapies for treating SCI.

## 2 Adrb2 Agonist Treatment Inhibits Microglia after SCI

**Figure 1:**
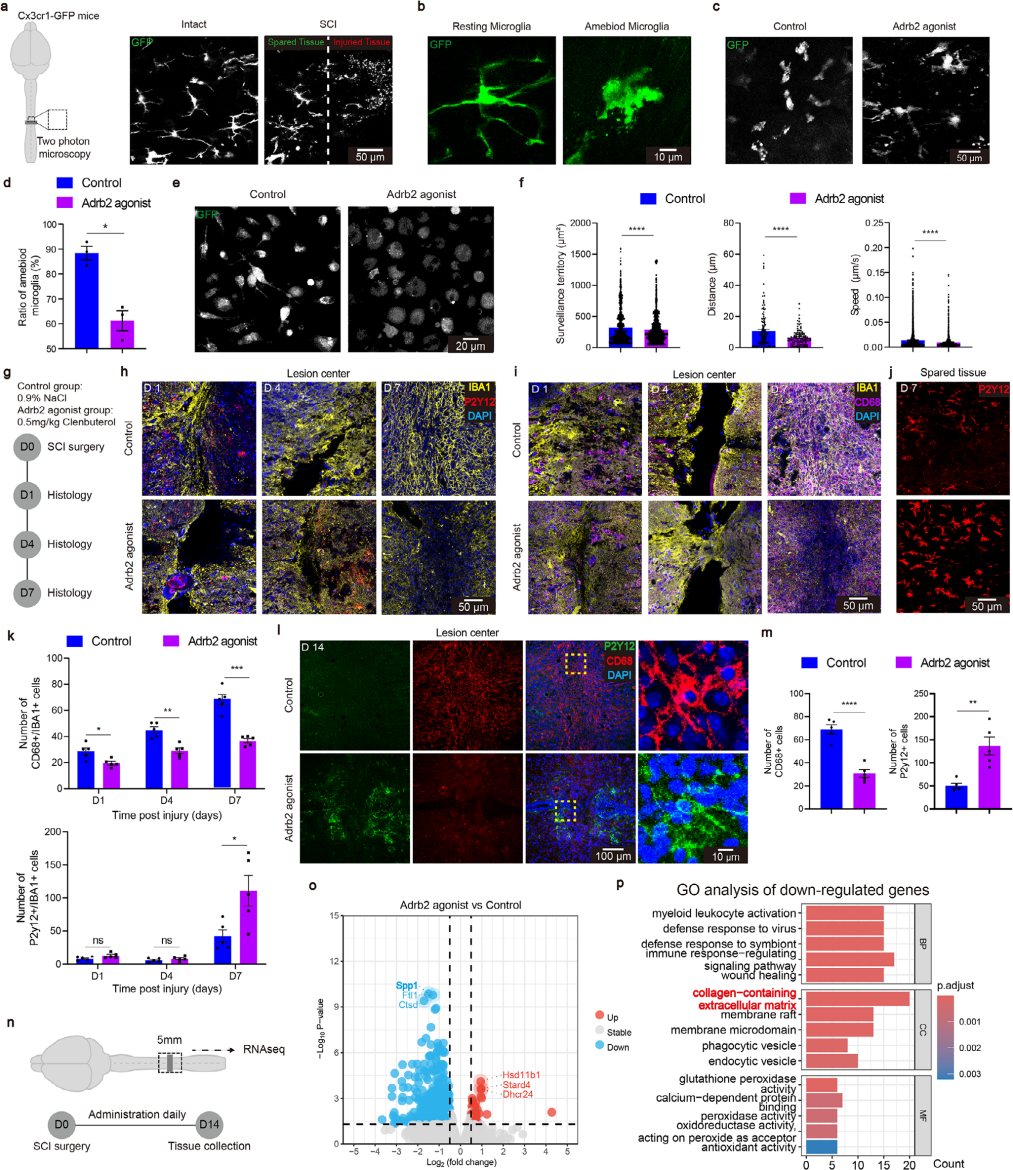
Adrb2 agonist inhibit microglial activity in SCI environment. a) Schematic diagram of the experimental design with microglial morphology before and after injury (0.5 mg/kg Adrb2 agonist was administered via intraperitoneal injection). b) Representative pictures showing morphological differences between resting and activated microglia. c) Representative sections showing the microglial activation. d) Quantification of microglial activation ratio (n = 3/3 mice). e) Representative showing macrophage morphology. f) Quantification of macrophage functional differences (cells were plated at a density of 2 × 10⁴ cells/well in 24‐well plates and treated with 100 ng/mL LPS for 24 h prior to analysis. Data represent from n = 3 independent cultures, with 3 technical replicates per condition). g) Schematic diagram of the experimental design for (h—k). h) Representative sections showing the activated states of microglia in lesion center. i) Representative sections showing the resting states of microglia in lesion center. j) Representative sections showing the resting states of microglia in spared neural tissue. k) Quantification of the CD68^+^ and P2Y12^+^ microglia at 1, 4, 7 dpi (n = 5/5 mice). l) Representative sections showing the activated and the resting states of microglia at 14 dpi. m) Quantification of the CD68^+^ and P2Y12^+^ microglia at 14 dpi (n = 5/5 mice). n) Schematic diagram of the experimental design for bulk RNA‐seq. o) Volcano plots of the injury group and the treatment group. p) GO analysis of the down regulated genes. ns *p* > 0.05, * *p* < 0.05, ** *p* < 0.01, *** *p* < 0.001, **** *p* < 0.0001. Two‐tailed unpaired *t*‐test (d, f, k, m). Data are shown as mean ±s.e.m.

Using two‐photon microscopy, was first examined the effects of an Adrb2 agonist, clenbuterol (0.5 mg kg^−1^, intraperitoneal injection), on microglial activity (**Figure** **1**a). In intact animals, consistent with previous observations in the brain,^[^ ^26^, ^27^ ^]^ treatment reduces microglial environmental surveillance capabilities (Extended Data Figure S1a‐c, Supporting Information). After a T10 complete spinal crush, in vivo imaging of Cx3cr1^GFP^ cells revealed that microglia transited from a homeostatic state to an activated amoeboid state (Figure 1b). At 1‐h post‐injury, a time point of robust microglial activation,^[^ ^40^ ^]^ more than 90% microglia were activated in vehicle treated controls, whereas about 40% remained non‐activated with Adrb2 agonist treatment (Figure 1c,d). Given that peripheral macrophages constitute a substantial fraction of injury‐site myeloid cells and contribute to secondary tissue damage and neuronal loss,^[^ ^20^, ^41^ ^]^ we further assessed the agonist's effects on macrophages in vitro (Figure 1e). Upon Lipopolysaccharide (LPS) stimulation, the Adrb2 agonist treatment significantly reduced macrophage surveillance territory, migration speed and distance over an observation period of 10 min (Figure 1f). These findings suggest that the Adrb2 agonist effectively inhibits the activation of both microglia and macrophage in the SCI microenvironment.

We went further to examine the effects of sustained Adrb2 agonist treatment on microglial states using CD68 and P2Y12 as markers for activated and resting microglia, respectively^[^ ^12^, ^42^ ^]^ (Figure 1g). Compared with controls, Adrb2 agonist treatment significantly reduced the number of CD68^+^ activated microglia in the lesion center at 1‐, 4‐, and 7‐days post‐injury (dpi) (Figure 1h, i, k). The treatment also increased the number of P2Y12^+^ resting microglia in spared neural tissues at 7 dpi (Figure 1j). Notably, after 14 days of Adrb2 agonist treatment, resting microglia were observed adjacent to the injury center, accompanied by reduced microglial activation within the lesion core (Figure 1l,m). Thus, Adrb2 agonist treatment promotes a transition of microglia toward a resting state, a prerequisite for scar‐free repair of the injured spinal cord.^[^ ^12^ ^]^

To examine how Adrb2 agonist treatment reshapes the scar transcriptome, we performed bulk RNA sequencing on lesion tissue (Figure 1n). After 2 weeks of treatment, 180 differentially expressed genes (DEGs) were identified between treated and control groups (49 upregulated, 131 downregulated; Figure 1o). Gene Ontology analysis of downregulated genes showed enrichment for pathways related to immune response and wound healing, as well as collagen/extracellular matrix organization and antioxidant activity (Figure 1p, Extended Data Figure S2a–c, Supporting Information). Notably, SPP1, a transcript closely associated with microglial activation,^[^ ^12^ ^]^ was significantly reduced, which we confirmed by immunofluorescence (Figure 1o, Extended Data Figure S2d,e, Supporting Information). These findings align with previous reports that Adrb2 agonist suppresses inflammation and protect neurons in CNS inflammatory diseases.^[^ ^7^, ^8^, ^9^ ^]^ Collectively, our data suggest that the Adrb2 agonist effectively inhibits microglial activation within the SCI microenvironment. Adrb2 agonist may be a novel pharmacological inhibitor for selective modulation of microglial activities following SCI.

## 3 Pharmacological Microglial Inhibition Remodels SCI Scar Microenvironment

**Figure 2:**
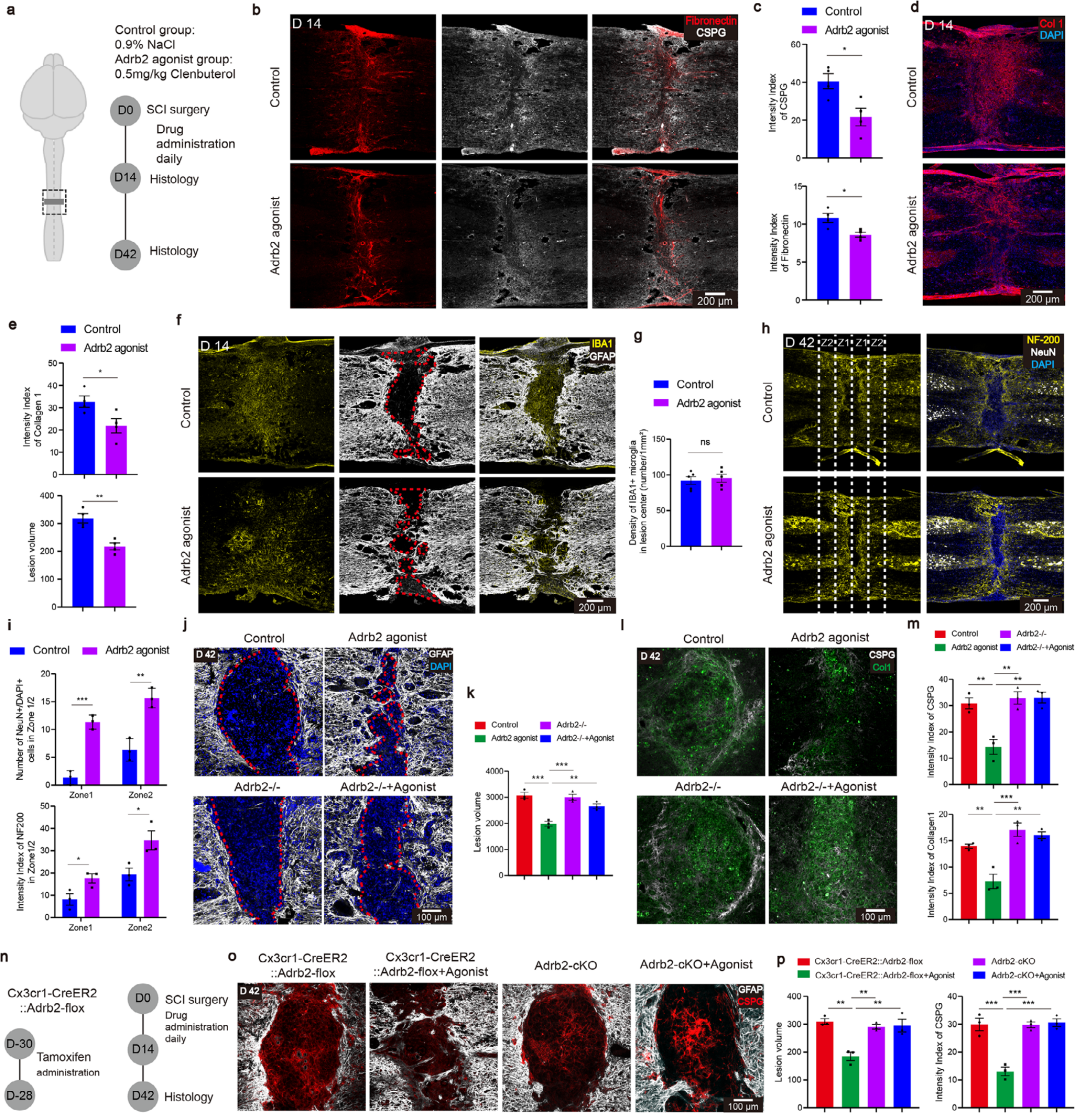
Microglial inhibition therapy remodels the scar microenvironment after SCI. a) Schematic diagram of the experimental design for microglial inhibition therapy. b) Representative sections showing the ECM. c) Quantification of the indicated immunoreactive intensity (n = 4/4 mice). d) Representative sections showing the fibrotic scar. e) Quantification of the Collagen I immunoreactive intensity and the lesion volume (n = 4/4 mice). f) Representative sections showing the spatial relationship between microglia and astrocyte. g) Quantification of the density of IBA1^+^ microglia in the lesion site (n = 5/5 mice). h) Representative sections showing NF‐200 and NeuN in different groups at 42 dpi. i) Quantification of the neuron number and NF‐200 immunoreactive intensity (n = 3/3 mice). j) Representative sections showing astrocyte scar in different groups at 42 dpi. k) Quantification of the lesion volume in different groups (n = 3/3/3/3 mice). l) Representative sections showing the ECM of the lesion site in different groups at 42 dpi. m) Quantification of the indicated immunoreactive intensity in the lesion site (n = 3/3/3/3 mice). n) Schematic diagram of the experimental design for Adrb2‐cKO. o) Representative sections showing the lesion site in different groups at 42 dpi. p) Quantification of the CSPG immunoreactive intensity and the lesion volume (n = 3/3/3/3 mice). ns *p* > 0.05, * *p* < 0.05, ** *p* < 0.01, *** *p* < 0.001. Two‐tailed unpaired *t*‐test (c, e, g, i). One‐way ANOVA, followed by post hoc Bonferroni correction (k, m, p). Data are shown as mean ±s.e.m.

Given the Adrb2 agonist‐induced transcriptional changes in scar tissue, we next investigated how microglial inhibition reshapes the scar microenvironment. After a 2‐week microglial inhibition (**Figure** **2**a), immunostaining revealed a significant reduction in the intensity of fibronectin deposition (Figure 2b,c). Moreover, the deposition of chondroitin sulfate proteoglycans (CSPGs), which are predominantly produced by astrocytes, was also markedly reduced at 14 dpi (Figure 2b,c). Decreased CSPG deposition may be attributable to promoted secondary activation of astrocytes driven by activated microglia.^[^ ^43^ ^]^ Collagen I (Col1), a marker of fibrotic scar,^[^ ^44^ ^]^ was likewise decreased, along with the reduction of overall fibrotic scar areas (Figure 2d,e). As these ECM are detrimental to neural regeneration, these findings implicate that microglial inhibition reduces inhibitory scarring.

The injury microenvironment of SCI mainly consists of astrocytic glial scar and fibrotic scar.^[^ ^5^ ^]^ Previous studies proposed that alterations in the scar microenvironment may affect the fate of scar‐forming astrocytes.^[^ ^6^, ^43^ ^]^ We thus examined the impact of microglial inhibition on astrocyte morphology. At 14 dpi, controls displayed a fully established scar, with astrocytes forming a distinct glial limitation at the lesion boundary (Figure 2f). In treated animals, astrocytes failed to form the characteristic compact glial scar structure (Figure 2f). Instead, they penetrated into the injury site to form bridge structures across the lesion, a morphological shift thought to support neural regeneration.^[^ ^45^, ^46^ ^]^ In addition, quantitative analysis of IBA1⁺ cells indicates that the treatment induces a phenotypic or functional shift rather than microglial depletion (Figure 2g).

The scar environment in SCI often leads to neuronal death and failure of axonal regeneration.^[^ ^47^ ^]^ To examine whether these environmental changes affect neuronal survival, we used NeuN staining to examine spared neurons and NF‐200 staining to visualize axons in different areas at the injury site (zone1: 0–200 µm, zone2: 200–400 µm) at 42 dpi (Figure 2h). Microglial inhibition resulted in greater numbers of spared neurons and axons in both regions (Figure 2i), suggesting that the remodeled scar environment by microglial inhibition supports neural survival and axonogenesis.

We next tested whether Adrb2 signaling mediates scar remodeling by using Adrb2^−/−^mice. In Adrb2^−/−^ mice, the Adrb2 agonist no longer reduced inhibitory scar formation or altered astrocyte morphology at 42 dpi (Figure 2j,k). No significant differences in Col1 and CSPGs deposition and scar area were observed between the treated and untreated groups in Adrb2^−/−^ group (Figure 2l,m). Notably, global Adrb2 knockout by itself does not affect scar formation and microglial morphology (Extended Data Figure S3a–f, Supporting Information), consistent with previous studies showing that microglial Adrb2 knockout does not alter microglial morphology, proliferation or stroke infarct size.^[^ ^48^ ^]^

To further test whether the Adrb2 agonist acts primarily on microglia, we crossed Cx3cr1^CreER2^ mice with Adrb2^flox^ mice. Following tamoxifen administration (Figure 2n), Adrb2 expression was specifically ablated in microglia (microglial Adrb2 conditional knockout, Adrb2‐cKO) (Extended Data Figure S4a–c, Supporting Information). In Adrb2‐cKO mice, the agonist no longer reduced inhibitory scar formation or CSPG deposition at 42 dpi (Figure 2o,p), mirroring the phenotype in Adrb2^−/−^ mice. This is consistent with the predominant expression of Adrb2 in microglia in the nervous system system.^[^ ^26^, ^27^ ^]^ Collectively, these results indicate that Adrb2 agonist regulates scar formation by activating Adrb2 signaling in microglia.

## 4 Pharmacological Microglial Inhibition Promotes Motor Function Recovery

**Figure 3:**
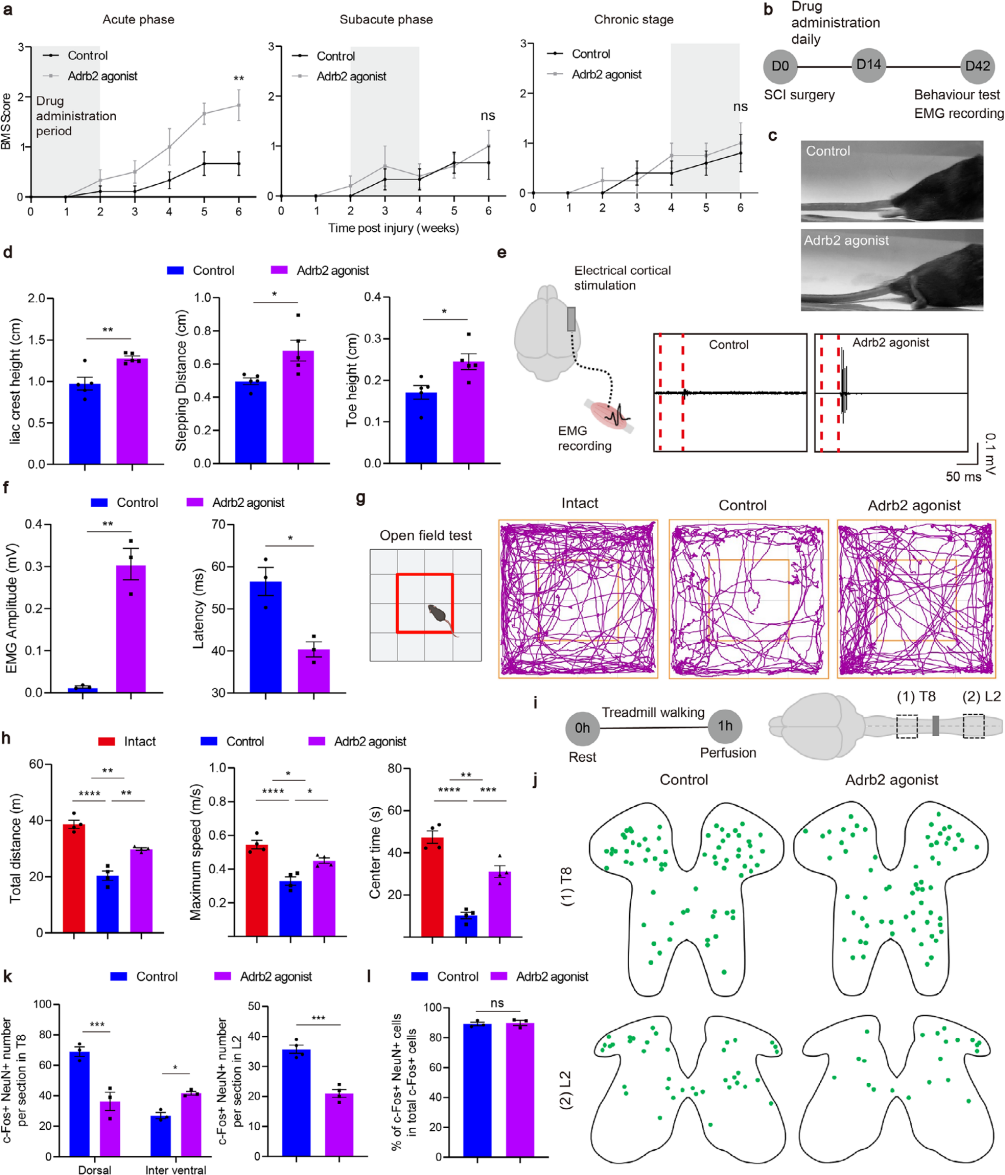
Microglial inhibition therapy promotes motor recovery and rebalances spinal cord circuits. a) BMS scores of the drug administration mice and control mice (n = 8/6;4/4;4/4 mice). b) Schematic diagram of the experimental design for testing. c) Representative pictures showing natural quadrupedal locomotion after acute phase treatment. d) Quantification of the iliac crest height, stepping distance and toe height (n = 5/5 mice). e) Representative EMG responses recorded in TA muscle evoked by cortical stimulations. f) Quantification of the amplitude and latency of the EMG signals (n = 3/3 mice). g) Trajectory of mice in open field chambers in different groups. h) Quantification of the total distance, maximum speed and center times in open field test (n = 4/4/4 mice). i) Schematic diagram of the experimental design for activating neurons. j) Representative sections showing c‐Fos^+^ NeuN^+^ cells in T8 sections and in L2 sections. k) Quantification of c‐Fos^+^ neurons in T8 sections (n = 3/3 mice) or in L2 sections (n = 4/4 mice). l) Quantification of c‐Fos^+^ NeuN^+^ cells in total c‐Fos^+^ cells (n = 3/3 mice). ns *p* > 0.05, * *p* < 0.05, ** *p* < 0.01, *** *p* < 0.001. Two‐tailed unpaired t‐test (d, f, k, l). One‐way ANOVA, followed by post hoc Bonferroni correction (h). Data are shown as mean ±s.e.m.

To evaluate the role of Adrb2 activation in motor recovery following SCI, we administrated the agonist for two‐week periods during three distinct post‐injury phases, the acute phase (0–14 dpi), subacute phase (14–28 dpi), and chronic phase (28–42 dpi), and measured locomotion recovery using the Basso Mouse Scale (BMS) scale^[^ ^49^ ^]^ (**Figure** **3**a). Significant motor recovery was observed only with treatment was initiated during the acute phase (Figure 3a), consistent with prior reports that Adrb2 agonists are most effective with early intervention.^[^ ^50^ ^]^ Accordingly, we employed the acute phase intervention protocol for subsequent in‐depth analysis (Figure 3b). Detailed kinematic analysis revealed significant improvements in iliac crest height, stepping distance, and toe height following pharmacological microglial inhibition (Figure 3c,d), indicating enhanced hindlimb weight‐bearing capacity and locomotor control.

We further evaluated supraspinal motor pathway integrity after Adrb2 agonist treatment. To this end, motor cortex electrical stimulation was applied with concomitant electromyographic (EMG) recordings from the tibialis anterior (TA) muscle at 42 dpi. Adrb2 agonist administration significantly increased EMG response amplitude and decreased the response latency (Figure 3e,f, Extended Data Figure S5a,b, Supporting Information), indicating enhanced connectivity between supraspinal centers and lumbar spinal circuits. Spontaneous locomotor activity was further assessed using the open field test (Figure 3g, Extended Data Figure S5c,d, Supporting Information). The treated animals exhibited significant increases in both total distance and maximum movement speed compared to controls (Figure 3h). Time spent in the central zone was markedly elevated as well (Figure 3h), potentially reflecting reduced anxiety levels secondary to motor function recovery.^[^ ^51^ ^]^

The restoration of the central pattern generator (CPG) function represents a critical determinant of motor performance recovery.^[^ ^52^, ^53^ ^]^ To evaluate the CPG functional states, spinal cord tissue was harvested following a standardized 1‐h treadmill walking protocol (Figure 3i).^[^ ^53^ ^]^ The co‐immunostaining of c‐Fos and NeuN revealed region‐specific alterations in c‐Fos^+^ neuronal density (Figure 3j). Treated animals exhibited reduction of c‐Fos^+^ neuron density within the thoracic dorsal horn (Figure 3k,l, Extended Data Figure S10a, Supporting Information). In addition, the quantitative analysis showed a significant decrease in c‐Fos^+^ neurons within the L2 segment (Figure 3j,k, Extended Data Figure S10b, Supporting Information). This shift in neuronal activation patterns suggests a transition from the aberrant firing states toward physiologically normative CPG activity.^[^ ^39^, ^53^, ^54^ ^]^ These findings substantiate microglial targeting as a fundamental therapeutic strategy for mitigating maladaptive plasticity post‐SCI.

**Figure 4:**
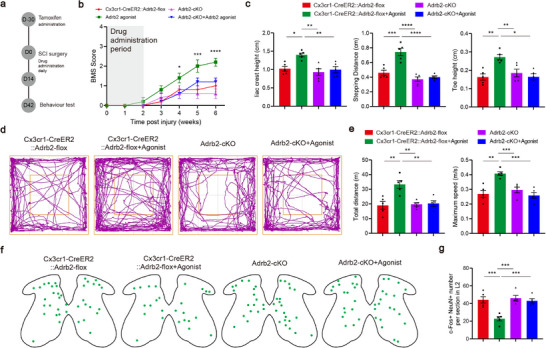
Adrb2 agonist mediate the promotion of motor functional recovery via acting on microglia. a) Schematic diagram of the experimental design for behaviour testing. b) Quantification of the BMS scores in different groups (n = 5/5/5/5 mice). c) Quantification of the iliac crest height, stepping distance and toe height (n = 5/5/5/5 mice). d) Trajectory of mice in open field chambers in different groups. e) Quantification of the total distance and maximum speed in open field test (n = 5/5/5/5 mice). f) Representative sections showing c‐Fos^+^ NeuN^+^ cells in L2 sections. g) Quantification of c‐Fos^+^ NeuN^+^ cells in L2 sections (n = 5/5/5/5 mice). * *p* < 0.05, ** *p* < 0.01, *** *p* < 0.001. One‐way ANOVA, followed by post hoc Bonferroni correction (c, e, g). Data are shown as mean ±s.e.m.

To investigate the mechanisms underlying Adrb2 agonist mediated motor recovery, we used microglia‐specific Adrb2‐cKO mice (**Figure** **4**a, Extended Data Figure S4d–f, Supporting Information). Adrb2‐cKO abrogated the pro‐recovery effects of Adrb2 agonist on BMS assessment (Figure 4b), failing to elicit restorative effects on iliac crest height, stepping distance, or toe height (Figure 4c). Consistently, therapeutic effects of Adrb2 agonist on open field performance and CPG function were abolished as well in Adrb2‐cKO mice (Figure 4d–g, Extended Data Figure S10c, Supporting Information). Notably, Adrb2‐cKO alone did not alter baseline motor performance or CPG activity. These data show that Adrb2 agonist–mediated functional recovery requires microglial Adrb2 signaling, supporting microglial modulation as the mechanism by which pharmacological intervention enhances motor recovery after SCI.

## 5 Scar Microenvironment Remodeling Supports Axon Regeneration Across the Injury Site

**Figure 5:**
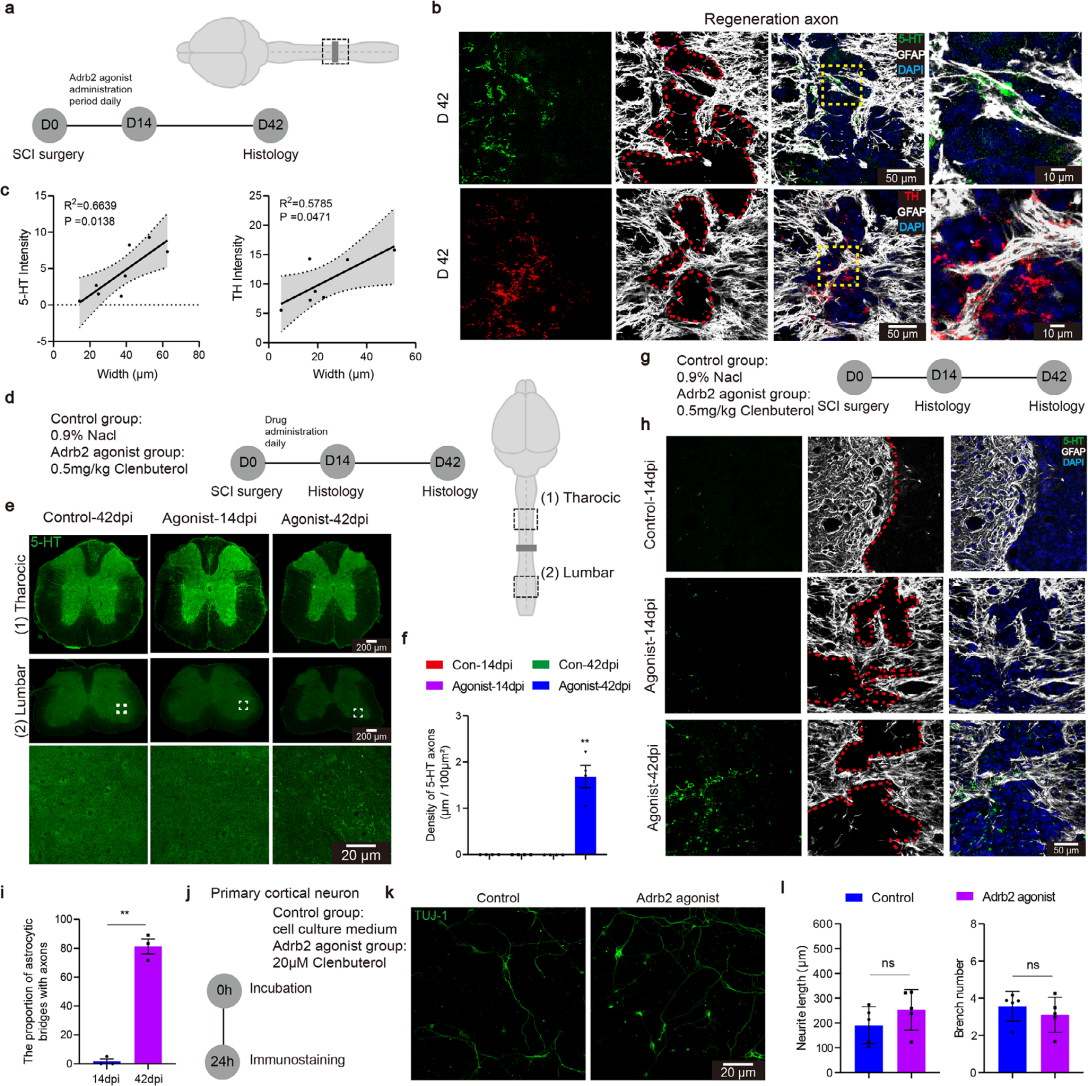
Microglial inhibition therapy protects supports axon regeneration cross the injury site. a) Schematic diagram of the experimental design for (b,c). b) Representative sections showing 5‐HT^+^ and TH^+^ axons traversing the bridge. c) Quantification of the correlation between 5‐HT/TH intensity and astrocyte bridge width (the points represent individual astrocyte bridges from 3 mice). d) Schematic diagram of the experimental design for (e,f). e) Representative sections showing the 5‐HT^+^ axons in lumbar segment. f) Quantification of the 5‐HT^+^ axons density in lumbar segment (n = 4/4/4/4 mice). g) Schematic diagram of the experimental design for (h,i). h) Representative sections showing astrocyte bridges with or without axons in different timepoint. i) Quantification of the proportion of astrocytic bridges with axons after treatment (n = 3/3 mice). j) Schematic diagram of the experimental design for neuronal culture. k) Representative sections showing the Tuj‐1 staining of primarily cultured cortical neurons. l) Quantification of neurite length and branch number of cultured cortical neurons (Data represent from n = 3 independent cultures, with 3 technical replicates per condition). ns *p* > 0.05, * *p* < 0.05, ** p < 0.01. Two‐tailed unpaired *t*‐test (i, l). One‐way ANOVA, followed by post hoc Bonferroni correction (f). Data are shown as mean ±s.e.m.

Axonal regeneration is a fundamental prerequisite for neural circuit reorganization and functional motor recovery.^[^ ^6^, ^52^, ^55^ ^]^ To determine whether microglial‐inhibition‐induced microenvironmental remodeling enables axonal traversal of the SCI lesion, we assessed regeneration dynamics of descending serotonergic (5‐HT) and tyrosine hydroxylase (TH) axons (**Figure** **5**a).^[^ ^56^ ^]^ Pharmacological microglial inhibition permitted both axonal populations to regenerate across lesion epicenters along the astrocyte‐derived bridging scaffold, and axonal density positively correlated with bridge width (Figure 5b,c). At 42 dpi, lesion regions devoid of astrocytic bridges exhibited complete failure of axonal regeneration (Figure 5b). Intriguingly, the width of astrocytic bridges remains unchanged at 14 and 42 dpi after treatment (Extended Data Figure S6a,b, Supporting Information), indicating that these growth‐promoting structures are established during the first two weeks post injury and remain stable thereafter. The integrity of complete SCI was demonstrated by the absence of 5‐HT^+^ axons in lumbar segments at 14 dpi in both control and treated cohorts (Figure 5d,e). By 42 dpi, robust 5‐HT^+^ axon regeneration was exclusively observed in Adrb2‐agonist treated animals (Figure 5f), indicating therapy‐facilitated axonal traversal across lesion.

To test whether Adrb2 agonist treatment has pro‐regenerative effects on descending axons, we assessed serotonergic axon regrowth across the lesion site at different time points after treatment (Figure 5g). At 14 dpi, descending axons remained distant from the lesion core (Figure 5h,i), consistent with previous studies showing that both regrowing ascending and descending axons require extended time period to approach and traverse the lesion site.^[^ ^57^, ^58^, ^59^ ^]^ Consistent with this, in vitro assay demonstrated no growth‐promoting effects of the Adrb2 agonist on axonal growth of primarily cultured cortical neurons (Figure 5j–l). Thus, Adrb2 agonist treatment promotes neural regeneration not through intrinsic neuronal mechanisms.

We next evaluated the temporal dependence of therapeutic intervention on scar microenvironment remodeling and axonal preservation. Initiating Adrb2‐agonist treatment at 14 or 28 dpi did not modify the established scar architecture and significantly inhibited axonal regeneration at lesion epicenters (Extended Data Figure S6c–f, Supporting Information). Anatomically, delayed treatment resulted in reduced astrocytic bridge formation, accompanied by robust astrogliosis and mature scar development (Extended Data Figure S6g, Supporting Information). This therapeutic limitation likely stems from maturation of fibrotic scar barriers by 14 dpi (Extended Data Figure S6c,e, h, Supporting Information), indicating that early intervention constitutes a critical temporal window for permissive scar transformation and astrocyte‐fate modulation.

To assess how Adrb2 expression post injury might influence pharmacologic efficacy, we employed a single‐cell RNA sequencing database spanning from pre‐injury conditions to 38 dpi (Extended Data Figure S7a,b, Supporting Information).^[^ ^60^ ^]^ Clustering using established cell markers^[^ ^20^, ^60^, ^61^ ^]^ showed that Adrb2 transcripts were predominantly detected in microglia across all stages, with a moderate reduction in the subacute stage and recovery at a chronic stage (Extended Data Figure S7c, Supporting Information). This dynamic was further validated by immunostaining (Extended Data Figure S7d,e, Supporting Information). Thus, the loss of therapeutic effect after 14 dpi is unlikely due to reduced Adrb2 expression. Taken together, our results indicate that pharmacological microglial inhibition promotes central axon regeneration by remodeling the injury environment at a time‐dependent manner, rather than through intrinsic neuronal mechanisms.

## 6 The ReST Undergoes Reorganization after Pharmacological Microglial Inhibition

**Figure 6:**
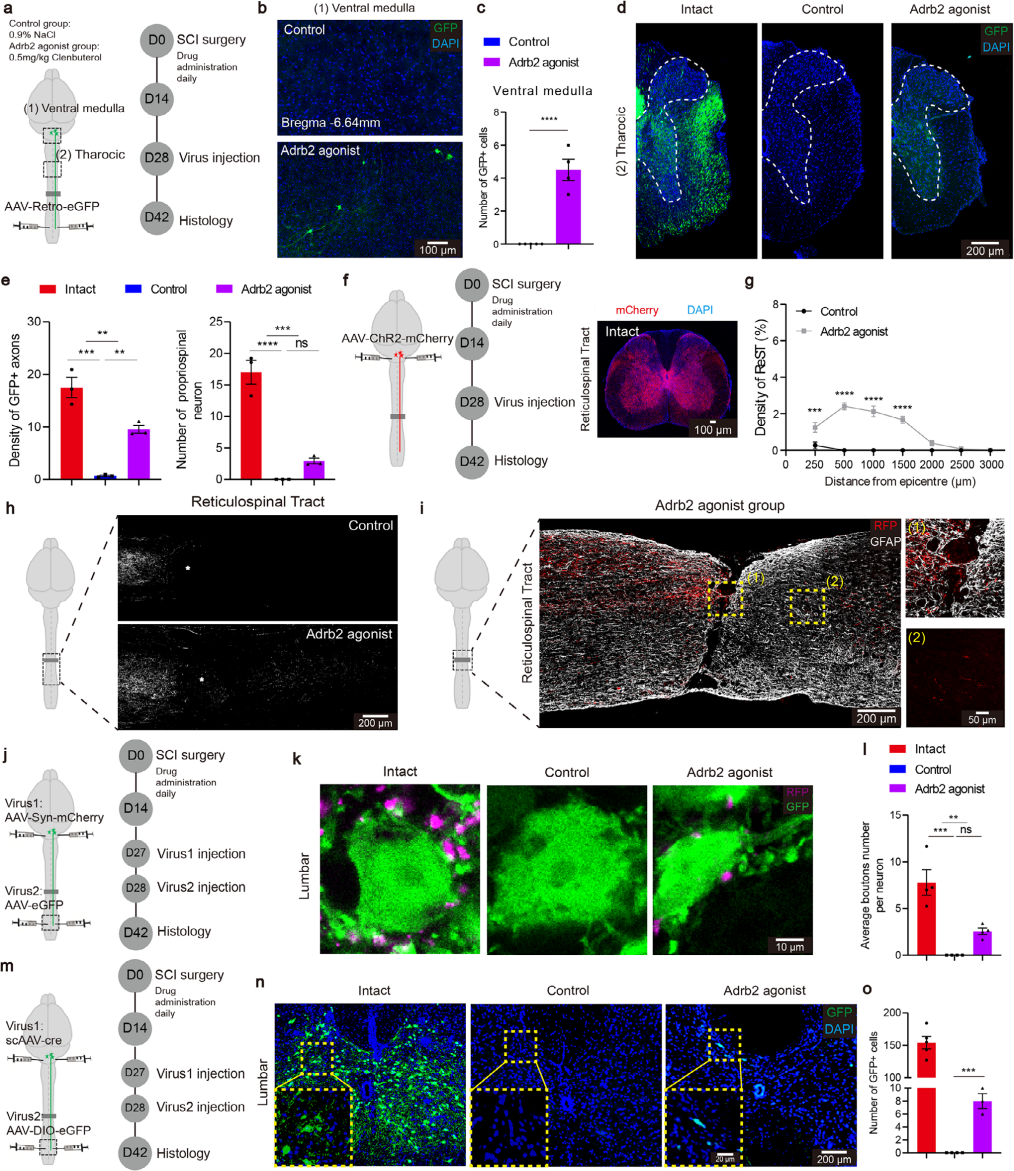
Microglial inhibition therapy aids reconstruction of the ReST after SCI. a) Schematic diagram of the experimental design for retrograde tracing. b) Representative sections showing the GFP^+^ neurons in ventral medulla. c) Quantification of the GFP^+^ neurons number in ventral medulla (n = 5/4 mice). d) Representative sections showing the GFP^+^ axons and neurons in thoracic segment. e) Quantification of the GFP^+^ axons density and neuron number in thoracic segment (n = 3/3/3 mice). f) Schematic diagram of the experimental design for anterograde tracing and tracing validation in intact spinal cord. g) Quantification of the density of ReST (n = 3/3 mice). h) Representative sections showing the projection of the ReST. i) Representative sections showing the astrocyte bridge support the ReST cross the injured site. j) Schematic diagram of the experimental design for labelling synapse. k) Representative sections showing the synaptic terminal in lumbar segment. l) Quantification of the synaptic terminal number in lumbar segment (n = 4/4/4 mice). m, Schematic diagram of the experimental design for labelling neurons. n) Representative sections showing the GFP^+^ neurons in lumbar segment. o) Quantification of the GFP^+^ neurons number in lumbar segment (n = 5/4/3 mice). ns *p* > 0.05, ** *p* < 0.01, *** p < 0.001, **** *p* < 0.0001. Two‐tailed unpaired *t*‐test (c). One‐way repeated‐measure ANOVA, followed by post hoc Bonferroni correction (e, l, o). Two‐way ANOVA, followed by post hoc Bonferroni correction (g). Data are shown as mean ±s.e.m.

To test whether descending motor tracts regenerate across the lesion after scar remodeling, we first injected a retrograde AAV into spinal segments caudal to the injury site (Figure 6a). Compared with controls, sparse GFP^+^ labeling was detected in propriospinal interneurons in thoracic segments and in the gigantocellular reticular nuclei of the ventral medulla, but was absent in layer V pyramidal neurons of the primary motor cortex (Figure 6b–e, Extended Data Figure S8a–c, Supporting Information), suggesting that regenerating axons derived from medulla grew into the denervated spinal cord.

Next, we performed anterograde tracing from the motor cortex and ventral medulla to assess whether regenerating tracts physically traverse lesion site within the remodeled microenvironments (Figure 6f, Extended Data Figure S8d, Supporting Information). Axonal tracing demonstrated ReST regeneration cross the lesion epicenters, whereas CST axons failed to penetrate scar borders (Figure 6g,h, Extended Data Figure S8e,f, Supporting Information). This tract‐specific regeneration pattern aligns with the differential regenerative competence among descending pathways.^[^ ^47^ ^]^ Importantly, GFAP co‐immunostaining confirmed that ReST axons crossing the SCI lesion were exclusively associated with the astrocyte‐derived bridge scaffold (Figure 6i). Thus, the permissive microenvironment modified by microglial inhibition may provide the structural foundation for circuit‐specific regeneration after SCI.

To examine whether regenerated ReST axons establish synaptic connectivity with downstream spinal neurons, we labeled ReST axons with pre‐synaptic mCherry (syp‐mCherry) and neurons in caudal segments with GFP (Figure 6j). In Adrb2 agonist treated animals, we observed a few pre‐synaptic boutons in close apposition to GFP^+^ somata (Figure 6k,l), consistent with ReST reinnervation of the denervated spinal cord. To confirm this further, we used a dual‐viral tracing system by injecting AAV1‐Cre into the ventral medulla, followed by intraspinal delivery of an AAV carrying Cre dependent GFP caudal to the lesion (Figure 6m). Because AAV1‐Cre mediates monosynaptic anterograde transmission,^[^ ^62^ ^]^ this approach allows specific labeling of neurons receiving direct ReST input. Only animals treated with the Adrb2 agonist, not controls, exhibited few GFP^+^ neurons in caudal thoracic segments, indicative of de novo synapse formation (Figure 6n,o). Taken together, these data indicate that, following microglial inhibition, regenerated ReST axons traverse SCI lesion epicenters via astrocyte‐derived bridges and form synapses with caudal spinal interneurons.

## 7 ReST Reorganization Mediates Motor Recovery after Pharmacological Microglial Inhibition

**Figure 7:**
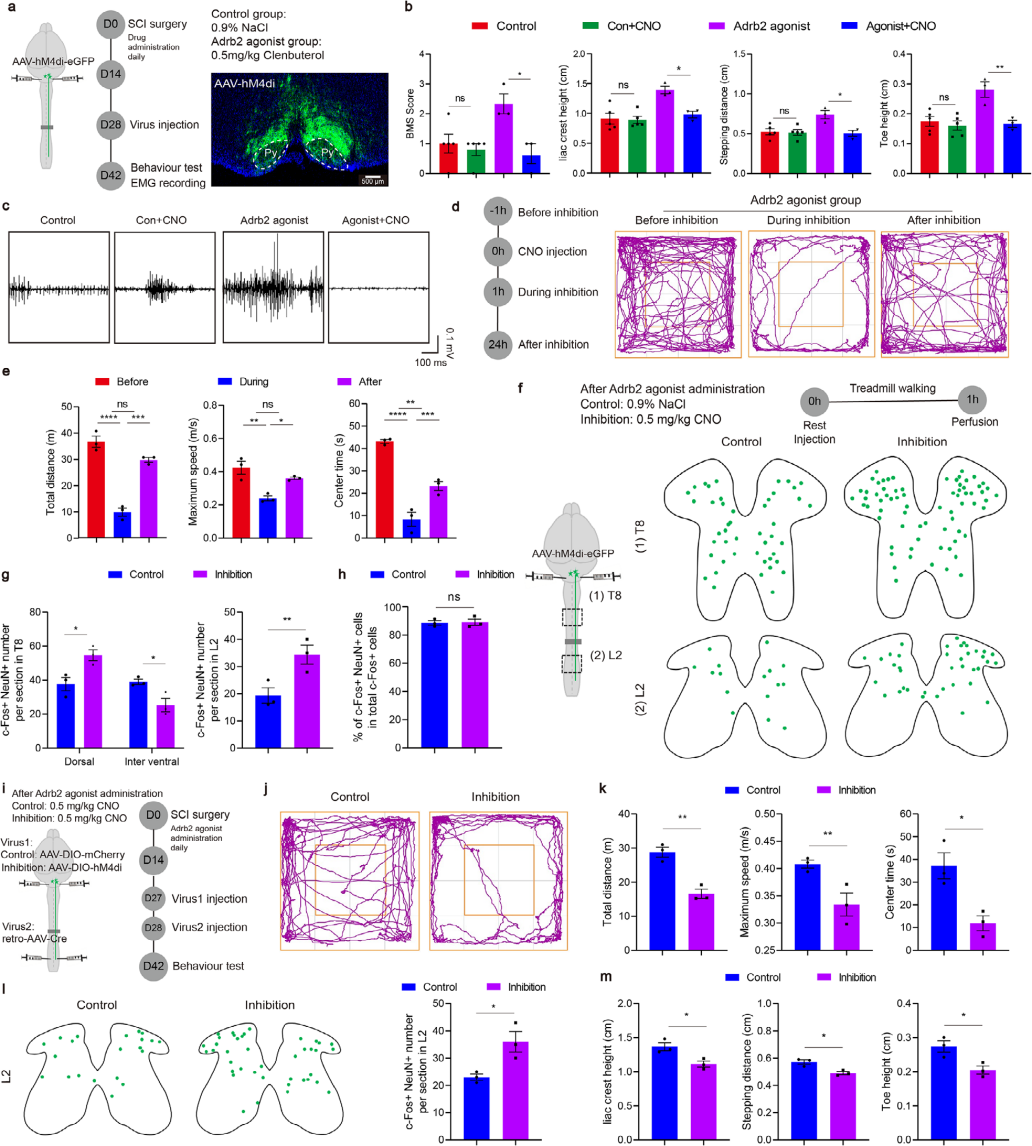
Reconstruction of the ReST contributed to the motor function recovery in microglial inhibition therapy. a) Schematic diagram of the experimental design for chemogenetic inhibition with the representative sections showing the expression of hM4di. Py, pyramidal tract. b) Quantification of the BMS score, iliac crest height, stepping distance and toe height (n = 5/3 mice). c) Representative EMG responses recorded in TA muscle during hindlimb locomotion. d) Schematic diagram of the experimental design for test and trajectory of mice in open field chambers before, during and after inhibition. e) Quantification of the total distance, maximum speed and center times in open field test (n = 3 mice). f) Schematic diagram of the experimental design and representative sections showing c‐Fos^+^ NeuN^+^ cells in T8 sections or in L2 sections. g) Quantification of c‐Fos^+^ NeuN^+^ cells in T8 sections or in L2 sections (n = 3/3 mice). h) Quantification of c‐Fos^+^ NeuN^+^ cells in total c‐Fos^+^ cells (n = 3/3 mice). i) Schematic diagram of the experimental design for selective chemogenetic inhibition. j) Trajectory of mice in open field chambers in different groups. k) Quantification of the total distance, maximum speed and center times in open field test (n = 3/3 mice). l) Representative sections showing c‐Fos^+^ neurons and quantification of c‐Fos^+^ neurons in L2 sections (n = 3/3 mice). m) Quantification of the iliac crest height, stepping distance and toe height (n = 3/3 mice). ns *p* > 0.05, * *p* < 0.05, ** *p* < 0.01, *** p < 0.001, **** *p* < 0.0001. Two‐tailed unpaired *t*‐test (b, g, h, k, l, m). One‐way ANOVA, followed by post hoc Bonferroni correction (e). Data are shown as mean ±s.e.m.

Because ReST reorganization is a recognized substrate for motor function recovery after SCI,^[^ ^37^ ^]^ we asked whether microglial inhibition restores function via the ReST by chemogenetically silencing gigantocellular neurons using hM4Di, the inhibitory Designer Receptors Exclusively Activated by Designer Drugs (DREADDs)^[^ ^63^ ^]^(**Figure** **7**a). In Adrb2‐agonist‐treated animals, selective silencing of reticulospinal neurons abolished the recovery of BMS scores, iliac crest height, stepping distance, and toe height, whereas control animals exhibited no significant alterations (Figure 7b). Furthermore, EMG demonstrated attenuated TA activation during locomotion following neuronal silencing (Figure 7c). In the open field test, chemogenetic inhibition reversibly suppressed total movement distance, maximum speed, and time spent in the central zone (Figure 7d,e). Importantly, once DREADD effects dissipated after 24 h post‐inhibition,^[^ ^64^ ^]^ all measures returned to pre‐inhibition baselines (Figure 7e). Thus, ventral medullary activation is required for microglial inhibition‐mediated recovery. Mechanistically, inhibition of reticulospinal neurons elevated c‐Fos activation in the thoracic and lumbar dorsal horn (Figure 7f–h, Extended Data Figure S10d,e, Supporting Information). This hyperactivity profile is consistent with loss of the restored locomotor circuit integrity achieved with microglial inhibition, suggesting that the ventral medulla dynamically regulates spinal circuit excitability.

To determine the role of regenerating ReST, we selectively inhibited ventral medullary neurons whose axons traverse the lesion sites (Figure 7i). Specific targeting was achieved by injecting AAV‐DIO‐hM4Di into the ventral medulla combined with thoracic and lumbar‐administration of AAVretro‐Cre. This axon‐regeneration specific silencing significantly compromised open field performance in mice with Adrb2 agonist treatment (Figure 7j,k). In contrast, thoracic propriospinal neuron‐specific inhibition did not alter the locomotion distance or maximum speed (Extended Data Figure S9a–c, Supporting Information). Consistently, only selective regenerating ReST inhibition abrogated the restoration of CPG function and motor recovery (Figure 7l,m, Extended Data Figure S9d–f, Supporting Information). Thus, ventral medullary inputs integrate into lumbar CPG circuitry via regenerated ReST axons, independent of propriospinal relay mechanisms. Collectively, our data identify the ReST as the principal neural substrate through which microglial inhibition achieves significant functional motor restoration.

## 8 Discussion

Our study demonstrates that Adrb2 agonist therapy suppresses microglial activity within SCI lesion sites and facilitates the microglia to a resting state. Importantly, Adrb2 agonist treatment initiated at the early stage reduces inhibitory ECM deposition, supports ReST axonal regeneration, re‐establishes functional synapses with caudal spinal circuits, and ultimately enhances motor recovery in SCI model. This microglial modulation strategy represents a dual‐mechanism therapeutic approach that concurrently attenuates scar formation and promotes circuit‐level neural repair. Such a strategy may have clinical potentials for comprehensive SCI rehabilitation and novel neurodegenerative disease interventions.

Following SCI, microglia exhibit complicated dual roles in spinal cord repairing. Early microglial activation promotes axonal debris phagocytosis and pro‐inflammatory cytokine release, which exacerbate secondary neurodegeneration and impair neuroregeneration.^[^ ^11^, ^41^ ^]^ Direct depletion of microglia, however, cannot achieve beneficial effects.^[^ ^19^, ^20^ ^]^ Recent research highlights that microglia have multiple protective functions in SCI, including wound constriction, lesion compaction, and coordination of multicellular interactions.^[^ ^19^, ^20^, ^21^ ^]^ Moreover, microglia secrete neuroprotective factors that can enhance neural regeneration in CNS injury models.^[^ ^65^, ^66^ ^]^ The functional duality of microglia necessitates balanced therapeutic strategies in SCI recovery.^[^ ^67^ ^]^ The strategy we used in the current study inhibits hyperactivated microglia without ablation them, resulting in decreased inflammation and reduced ECM deposition yet maintained wound‐healing capacities.

In our study, pharmacologic activation of microglial Adrb2 reduced inhibitory scar features and altered astrocyte morphology, and these effects were absent in microglia‐specific Adrb2 knockout, indicating receptor dependence. A plausible mechanism is that β2‐adrenergic signaling in microglia dampens NF‐κB‐associated inflammatory cues (e.g., IL‐1α, TNF, C1q), which could in turn reduce astrocyte reactivity and CSPG/collagen I deposition.^[^ ^43^, ^68^ ^]^ To fully explore the underlying mechanisms, future studies that integrate single‐cell sequencing and cell type‐specific Cre lines are needed to dissect the distinct contributions of microglia in modulating other scar‐forming cells and infiltrating immune populations at different stages after SCI.

While emerging scar‐targeting strategies inhibit fibrotic deposition and promote axonal growth,^[^ ^31^, ^32^, ^69^ ^]^ the specific supraspinal pathways regaining control of caudal circuits remain unclear.^[^ ^34^, ^70^ ^]^ ReST exhibits exceptional post‐injury plasticity which facilitates locomotor recovery and fine motor control.^[^ ^71^, ^72^, ^73^ ^]^ In our model, ReST demonstrated enhanced regenerative competence within the modulated environments. Future studies may evaluate combinatorial microglial inhibition and neuromodulation strategies.

Neuronal hyperactivation is common across denervated spinal segments. For instance, SCI drives overactivation of T8 dorsal horn neurons, causing aberrant sensory and autonomic network hyperactivity that induces systemic immunosuppression and predisposes to life‐threatening infections.^[^ ^74^, ^75^ ^]^ Notably, post‐SCI microglial depletion prevented maladaptive synaptic and structural plasticity within autonomic networks.^[^ ^76^ ^]^ In our study, pharmacological microglial inhibition recapitulated this neuroprotective effect, significantly reducing c‐Fos^+^ neuronal density in the T8 dorsal horn. Because CPG dysfunction significantly contributes to motor impairment after severe SCI,^[^ ^52^, ^53^ ^]^ the reduced c‐Fos activity we observed in lumbar segments, coinciding with regenerated ReST reinnervation, suggests a shift of dysfunctional CPG networks toward more physiological states. The mechanism underlying ReST‐mediated CPG normalization warrants further investigation, potentially involving restoration of local excitatory‐inhibitory balance.^[^ ^77^, ^78^ ^]^

Chemotropic guidance combined with pro‐regenerative molecules can drive targeted reinnervation of specific propriospinal neuron subpopulations to natural target regions and achieve significant functional restoration.^[^ ^70^ ^]^ In our study, propriospinal circuit remodeling did not significantly contribute to therapy‐mediated motor recovery, likely reflecting the restricted regenerative competence of discrete propriospinal subtypes capable of supporting functional repair.^[^ ^79^ ^]^ While environmental remodeling establishes an essential permissive substrate for propriospinal axon reinnervation, its supportive effects remain non‐selective across neuronal subpopulations. Future studies should evaluate the combinatorial strategies integrating scar‐modifying therapies with subtype‐specific regeneration paradigms to enhance functional recovery.^[^ ^80^, ^81^ ^]^

**Figure 8:**
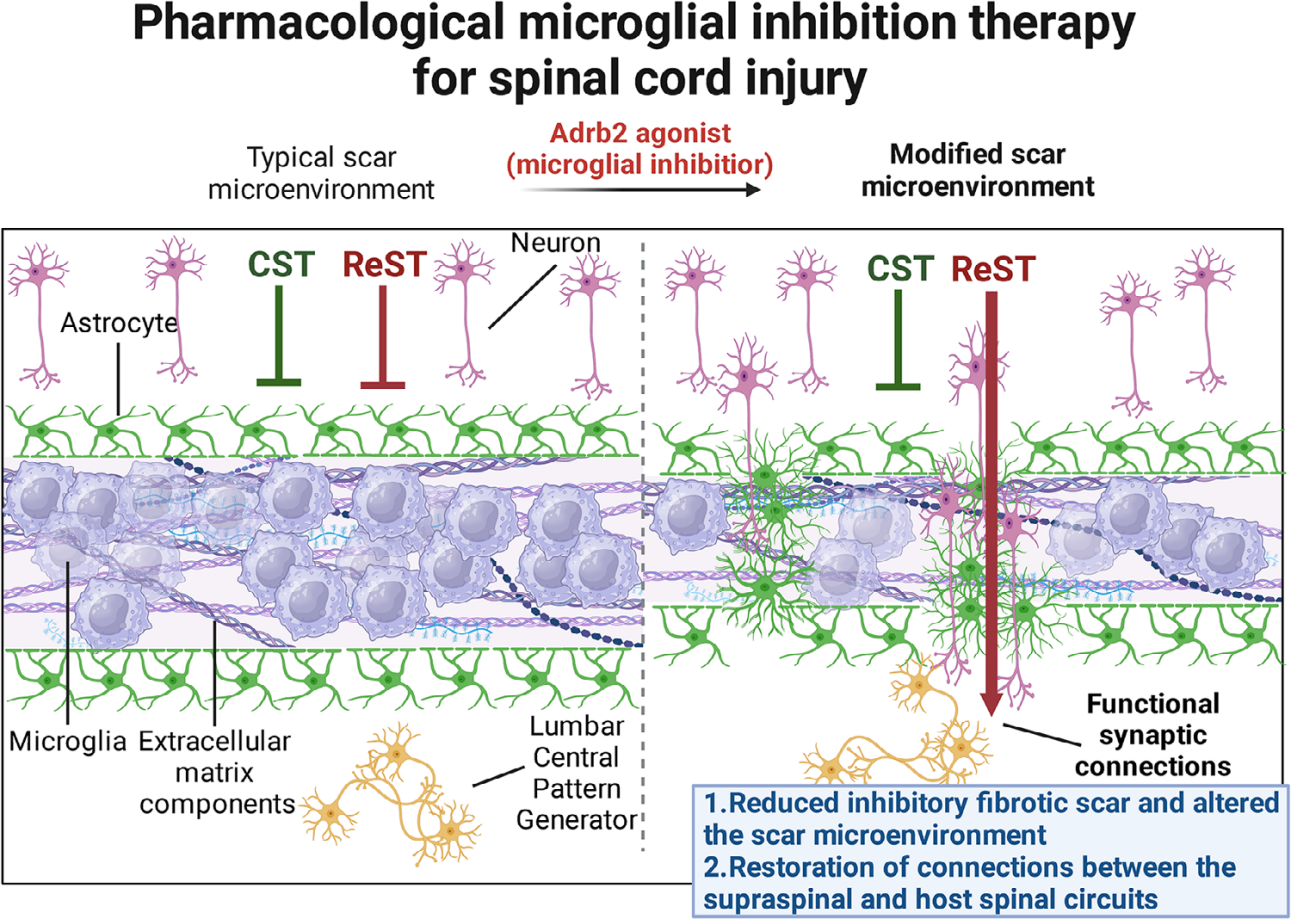
Schematic diagram of microglial inhibition therapy. Adrb2 agonist administration post‐SCI suppresses microglial activation, reduces ECM deposition within the scar microenvironment, and attenuates reactive astrogliosis. This permissive microenvironmental remodeling facilitates ReST axonal regeneration across lesion epicenters, reconstructs functional synapses with the caudal spinal circuits, and ultimately restores motor function.

In summary, we established a scar microenvironment remodeling therapy via microglial inhibition and identified the ReST‐mediated neural circuit underlying functional recovery (**Figure** **8**). This work advances SCI scar‐targeting paradigms and provides a mechanistic framework for integrating glial modulation with neuromodulation therapies.

## 9 Experimental Section

### Mice

All experimental protocols were reviewed and approved by the First Affiliated Hospital, Zhejiang University (Ethical Approval No. 2021–092). The experimental animals included C57BL/6 wild‐type (Vital River Laboratory, China), Cx3cr1^GFP^ (Jackson Laboratory, USA), Cx3cr1^CreERT2^ (Jackson Laboratory, USA), Adrb2^flox^ (Cyagen, China) and Adrb2^−/−^ (Cyagen, China) strains. Female C57BL/6 mice (postnatal week 8, bodyweight 18–20 g) were housed under controlled conditions (temperature: 22±1 °C; humidity: 55±5%) within a specific pathogen‐free (SPF) facility. Mice were maintained under a standardized light‐dark cycle with free access to autoclaved chow/water ad libitum.

### SCI Modelling and Post‐Surgical Care

A T10 spinal cord crush injury model was performed using standardized surgical procedures.^[^ ^12^ ^]^ A midline incision was used to expose T9‐T11 laminae and perform laminectomy. After the T10 spinal cord was exposed, a delicate 0.1‐mm No. 5 Dumont micro‐forceps (Switzerland) was then used to crush the full width of the spinal cord. The instrument was used with controlled pressure to ensure a complete, 360‐degree disruption of target tissues. The spinal cord was then precisely compressed for 3 s under careful observation to create a uniform injury.

1 mL of saline solution was subcutaneously injected to maintain fluid balance after the myofascial layers were closed. Core body temperature was maintained using thermoregulated recovery pads until the mice were fully conscious. Analgesia was managed with 0.05 mg kg^−1^ of buprenorphine daily for 3 days, along with bladder expression (twice per day) to prevent urinary retention. The mice were sacrificed when the body weight loss >15%.

### Immunohistochemistry

After terminal anesthesia, mice were transcardially perfused with saline, followed by perfusion with 4% paraformaldehyde (PFA). The PFA‐fixed specimens underwent cryoprotective equilibration in 30% sucrose solution at 4 °C for 24–48 h until osmotic equilibrium was achieved. Tissues were subsequently cryoembedded in hydrophilic polymer matrix (Tissue‐Tek OCT Compound, Sakura Finetek, USA) and stored at under −80 °C for cryosection.

Coronal and sagittal sections (spinal cord or brain, 40 µm) were cryosectioned and cryopreserved at −20 °C in antifreeze solution until immunohistochemical processing. Following PBS rehydration, antigen retrieval was performed through 10‐min permeabilization in 0.5% Triton X‐100. The non‐specific binding sites were subsequently blocked via 60‐min incubation in 10% donkey serum (Jackson ImmunoResearch, USA) supplemented with 0.2% Triton X‐100 in PBS at ambient temperature.

The primary antibodies included: mouse anti‐GFAP (Abcam(ab4648), 1:1000, UK); rabbit anti‐GFAP(Affinity(DF6040), 1:1000, USA); goat anti‐5‐HT (Immunostar(20 080), 1:5000, USA); mouse anti‐IBA1 (Santa Cruz(sc‐32725), 1:100, USA);mouse anti‐IBA1 (ABclonal(A27316), 1:1000, China); rabbit anti‐RFP (Abcam(ab34771), 1:1000, UK); rabbit anti‐P2Y12 (Abcam (Ab300140), 1:1000, UK); rabbit anti‐CD68 (ABclonal (A6554)), 1:1000, China); mouse anti‐CD68 (Abcam (ab201340), 1:500, UK); rabbit anti‐fibronectin (Millipore(AB2033), 1:1000, Germany); rabbit anti‐collagen I (Abcam(ab21286), 1:1000, UK); mouse anti‐CSPG (Sigma‐Aldrich(C8035), 1:1000, USA); chicken anti‐GFP (Abcam(ab13970),1:1000, UK); chicken anti‐TH (Abcam (ab76442), 1:1000, UK); mouse anti‐Tuj‐1 (Biolegend (801 202), 1:1000, USA); mouse anti‐NeuN (Millipore(MAB377), 1:800, Germany); rabbit anti‐c‐Fos (Cell Signaling(2250s), 1:800, USA); rabbit anti‐NF‐200 (Abcam(ab207176), 1:1000, UK); rabbit anti‐Adrb2 (Abcam(ab182136), 1:500, UK); rabbit anti‐SPP‐1 (GenuIN (50 655), 1:500, China).

Secondary antibodies included: Alexa Fluor 488‐conjugated donkey anti‐chicken /goat/rabbit/mouse (A‐11008/A‐11055/A‐11001/A‐11039), Alexa Fluor 555‐conjugated donkey anti‐rabbit/mouse (A‐32732/A‐32727), and Alexa Fluor 647‐conjugated donkey anti‐rabbit (A‐31573). All species‐specific secondary antibodies were commercially sourced from Invitrogen (Thermo Fisher Scientific, USA).

Primary antibodies were applied in a 16‐h 4 °C incubation protocol, followed by species‐matched secondary antibody conjugation (3 h, 4 °C). Nuclear counterstaining was performed using DAPI (Beyotime, China) under ambient conditions (10 min). Spinal cord and brain sections were imaged via high‐resolution confocal microscopy (Leica SP8, Germany).

### Bulk‐Seq

At the endpoint of 14‐day drug intervention, mice were perfused with saline under anesthesia, and a 5 mm section of spinal cord tissue surrounding the lesion site was collected and stored at −80 °C.^[^ ^60^ ^]^ The samples were processed using AccuraCode lysis buffer and subjected to Illumina sequencing with the assistance of Singleron (China). Differentially expressed genes (DEGs) were conducted through DESeq2 (0.5‐fold change in expression and a p‐value < 0.05 between the control and treatment groups). DEGs were visualized using ggplot2 and heatmap packages in the form of volcano plots and heatmaps. GO functional enrichment analysis of the down regulated genes (defined by a fold change >1 and p‐value < 0.05) was performed by the clusterProfiler package.

### Behavioral Tests

Motor function was evaluated using the BMS rating scale, with weekly assessments conducted following established protocols.^[^ ^49^, ^53^ ^]^ Parameters were quantified as follows: ankle joint angular displacement ≤50% of the total range of motion (ROM) was classified as restricted arthrokinesis (BMS 1.0), whereas displacement >50% ROM indicated unrestricted arthrokinesis (BMS 2.0). Gait patterns were categorized based on weight‐supporting abilities: intermittent dorsal paw loading during partial stepping cycles was defined as dorsiflexion‐dominant ambulation (BMS 3.0).^[^ ^53^ ^]^ Bilateral hindlimb performance was scored according to the maximal functional capacity observed in either limb.

Ground locomotion kinematics, including iliac crest height, stepping distance and toe height, were analyzed on a linear runway according to established protocols.^[^ ^82^ ^]^ Anatomical landmarks were identified using a dermatographic pen for motion‐tracking. A 60 Hz imaging system (GoPro, USA) was utilized to capture 10–15 consecutive gait cycles per subject for quantitative analysis. All behavioral assessments and subsequent data interpretations were performed by an investigator who was blinded to experimental protocols.

### Open Field Test

The open field apparatus consists of a central square zone (250 × 250 mm) and a surrounding peripheral zone in a square box (500 × 500 × 500 mm). Mice were placed in the central zone of the open field apparatus and allowed to acclimate freely for 10 min. After acclimation, their spontaneous movements were recorded for 10 min. Behavioral parameters, including total distance traveled, maximum movement speed, and time spent in the central zone, were analyzed using Anymaze behavioral software (Stoelting, USA).

### Stereotactic Viral Injection

The experimental procedures were adapted from our previous work.^[^ ^53^ ^]^ Mice were anesthetized via inhaling 1.5–2% isoflurane/O_2_ vapor mixture and surgically immobilized in a precision stereotaxic frame using maxillary restraint and dual aural fixation cannulae to achieve craniocerebral stabilization.

For the CST tracing experiment, bilateral sensorimotor cortex was exposed via precise craniectomy, and glass micropipettes connected to an injection pump were used to deliver AAV2/8‐hSyn‐ChR2‐mcherry at stereotaxic coordinates relative to bregma: anteroposterior +1.0 to −1.5 mm in 0.5 mm increments; mediolateral ±1.3 mm; dorsoventral 0.6 mm from pial surface (12 delivery sites, 100 nL site^−1^). Viral microinfusions were executed at a controlled rate of 20 nL min^−1^, with a 5‐min dwell time to ensure post‐injection diffusion equilibrium. Anterograde neural tracing of the ReST and chemogenetic silencing via hM4Di were achieved through stereotactic navigation of glass micropipettes across bilateral cerebellar craniectomy windows. AAV2/8‐hSyn‐ChR2‐mcherry, AAV2/9‐hSyn‐hM4di‐eGFP, AAV2/9‐hSyn‐DIO‐mcherry, AAV2/9‐hSyn‐DIO‐hM4di‐eGFP, AAV2/9‐hSyn‐Synaptophysin‐mCherry, and scAAV1/2‐hSyn‐cre were delivered to the ventral medulla at stereotaxic coordinates relative to bregma: anteroposterior −4.6 to −4.8 mm; mediolateral ±0.3 mm; dorsoventral 4.6 mm from pial surface (4 delivery sites, 100 nL site^−1^).

For intraspinal microinjection, AAV2/retro‐CAG‐eGFP, AAV2/retro‐hSyn‐Cre, AAV2/9‐hSyn‐GFP and AAV2/9‐hSyn‐DIO‐eGFP were injected into bilateral lumbar segments 2–4 or thoracic segments 6–8. Stereotaxic delivery coordinates were established at bilateral 0.5‐mm relative to the midsagittal suture, comprising three rostrocaudal points spaced at 1‐mm intervals. Each points underwent dual‐depth administration (dorsoventral: 0.5 mm and 1.0 mm from pial surface) via nanoinjector‐controlled microinfusions (12 total delivery sites; 100 nL site^−1^). AAV vectors were standardized to 1–2 × 10^13^ copies mL^−1^.

All recombinant viral constructs used in this study were commercially purchased from BrainVTA (China). The virus will be injected 2 weeks prior to the endpoint to ensure adequate expression levels.

### Compound Treatment

To induce Adrb2 activation, 0.5 mg kg^−1^ clenbuterol (MedChemExpress, USA) was administered daily via intraperitoneal injection for 2 weeks. Pharmacological activation of Gi‐coupled DREADD receptors was initiated through systemic administration of 0.5 mg kg^−1^ CNO (Enzo Life Sciences, USA). Intraperitoneal delivery was timed at 14 days post‐ AAV injection to ensure optimal receptor expression. The control group received daily injections of 0.9% NaCl.

For Adrb2 activation, during the treatment period, the Adrb2 agonist was administered daily via intraperitoneal injection at a dosage of 0.5 mg kg^−1^. Behavioral tests and tissue collection were performed 24 h after administration of clenbuterol. For hM4di inhibition, behavioral assessments were conducted 1 h before (before inhibition), within 1 h (during inhibition) or 24 h (after inhibition) after systemic administration of CNO.

For Cre recombination induction in mice, 100 mg kg^−1^ of tamoxifen (Sigma, USA) dissolved in corn oil (Sigma, USA) was administered via intraperitoneal injection for three consecutive days.

### Cortical Stimulation and EMG Recording

Following previously established methods,^[^ ^53^ ^]^ experimental stability was ensured by rigid immobilization of the cephalic fixation plate to eliminate cranial motion artifacts. Epidural stimulating electrodes were stereotactically positioned over the right motor cortical hindlimb representation area at the following stereotaxic coordinates relative to bregma: anteroposterior 0–1.5 mm, mediolateral 0.5–1.5 mm. Neuromuscular recordings were obtained using a bipolar EMG electrode inserted percutaneously into the TA muscle belly of the ipsilateral hindlimb via a 30‐gauge hypodermic needle. A subcutaneous reference electrode was implanted in the thoracolumbar region.

Cortical activation was achieved using a programmable stimulator‐isolator system (A.M.P.I., USA) to deliver biphasic current pulses (0.2 ms pulse, 100 ms, 20 Hz frequency, 0.5–1.5 mA intensity) to awake, quadrupedally positioned mice. Evoked electromyographic responses from the TA muscle were quantified by peak‐to‐peak amplitude analysis following corticomotor pathway activation. Signal acquisition utilized a differential amplification system (A‐M Systems, USA) with bandpass filtering (10–1000 Hz), synchronized to a 16‐channel data acquisition unit (AD Instruments, New Zealand) sampling at 4 kHz. Post hoc signal processing and quantification were performed using LabChart 8 software (ADInstruments, New Zealand) with standardized event detection algorithms.

### Cell Culture and Experiments

For the BMDM cell experiments, bone marrow cells were aseptically flushed from the tibiae of Cx3cr1‐GFP transgenic mice, and cultured in DMEM (Gibco, USA) in the presence of recombinant mouse M‐CSF (40 ng mL^−1^, Sigma‐Aldrich, Germany) at 32 °C. Primary cortical neuronal cultures were established from C57BL/6J mouse embryos at embryonic days 13.5–14.5 (E13.5–E14.5). Under stereomicroscopic guidance, brain structures were microdissected, and tissue was dissociated enzymatically‐mechanically via digestion.^[^ ^83^ ^]^ Neural cell suspensions were seeded onto 15‐mm coverslips within 12‐well culture plates. After 72 h in vitro (post‐attachment), cultures were treated with either neurobasal medium or 20 µM clenbuterol hydrochloride in neurobasal medium. Following a 24‐h pharmacological intervention, cells were fixed in 4% PFA for immunocytochemical analysis using Tuj‐1 as a neuronal marker.

### Microglia and Macrophage Two‐Photon Imaging

For in vivo microglial imaging, a midline dorsal surgical approach was made at the T9‐T11 spinal segment to perform laminectomy and achieve epidural access. Hydrogel was applied to the exposed area throughout the experiment. The open laminectomy window was covered with a small coverslip. After the T10 crush injury was performed, the mice were transferred to a microscope (Leica SP8 Multiphoton Scanning Microscope with DIVE Multiphoton, Germany) for imaging, as maintained under anesthesia. Body temperature was kept at 37 °C using a thermostatic heating system. Microglia (eGFP‐labeled, 500–550 nm) were visualized using a laser with an excitation wavelength of 880 nm. Images were acquired at a depth of at least 50 µm, with vertebrae stabilized using ear bars to exclude any surgical artifacts. Z‐stack images were captured with a 3 µm step size at a resolution of 512 × 512 pixels to observe the effects of the drug on microglial dynamics. Imaging was repeated 60 min later to assess differences in microglial responses to injury.

For in vitro visualization of BMDM cells, BMDM cells were plated at a density of 2 × 10⁴ cells well^−1^ in 24‐well plates. Cells were treated with LPS (100 ng mL^−1^) for 24 h prior to analysis. Next, BMDM cells were cultured with 20 µM clenbuterol and imaged at a resolution of 512 × 512 pixels every 30 s for 10 min to monitor the dynamic effects of the drug on BMDM cells. Tracking of BMDM cells were conducted using the Imaris surfaces function for detailed cellular analysis. The microglial response at t1 time point (R(t1)) is calculated as R(t1) = Rx(t1)/Ry(t0).

### ScRNA‐Seq Data Analysis

The single‐cell transcriptomic profiles of the spinal cord were sourced from the Cheng Liming's laboratory (https://doi.org/10.6084/m9.figshare.17702045).^[^ ^61^ ^]^ The dataset comprises 10‐mm spinal cord segments centered on lesion epicenters, obtained through a longitudinal sampling regimen spanning pre‐injury baseline through 38 dpi. Downstream analysis was performed using Seurat v5,^[^ ^84^ ^]^ with quality control filters applied to retain cells exhibiting >500 feature counts, <5000 RNA counts, and mitochondrial gene expression levels below 10%. Normalization was implemented via the LogNormalize method with a scaling factor of 10 000. Highly variable genes were algorithmically defined by the FindVariableFeatures module, retaining the top 5000 features based on dispersion metrics. High‐dimensional transcriptomic data reduction and cellular taxonomy resolution were implemented using Uniform Manifold Approximation and Projection (UMAP), a nonlinear manifold learning algorithm optimized for preserving global data topology. Cluster‐ DEGs were found using the FindAllMarkers. Data visualization workflows were executed through the singlecellpepline (SCP, https://github.com/zhanghao‐njmu/SCP) computational framework.

### Quantification

Immunofluorescence signal quantification and normalization within the lesional microenvironment was performed using ImageJ (1.54p, NIH, USA).^[^ ^38^ ^]^ For testing the differences in macrophage functions, the speed and range of the cells are measured every 30 s (a total of 20 times within 10 min), and the total distance traveled in 10 min was acquired. The extent of the injury scar was measured in GFAP or Collagen1 staining. For axonal density at the spinal cord segments, the length of axons within the specified area was quantified. For axonal density within astrocytic bridges, the fluorescence intensity of axons inside the bridges was quantified. For anterograde tracing of the ReST and CST, the axon density index was represented as the ratio of fluorescence intensity to different distances. To avoid the impact of variations in spinal cord width on the lesion area in mice, we normalized the lesion area by individual spinal cord widths.

### Statistical Analysis

Statistical analyses were performed using GraphPad Prism (v8.0, USA). Two‐tailed unpaired *t*‐test, one‐way or two‐way analysis of variance (ANOVA) were used as appropriate. Bonferroni correction was applied to adjust the p‐value for multiple comparisons. All continuous variables were expressed as mean ± standard error of the mean (SEM). Statistical significance thresholds were stratified as follows: ****p < 0.0001; ***p < 0.001; **p < 0.01; *p < 0.05; NS (not significant, *p* ≥ 0.05). Perform post hoc power analyses on the data using the PWR package and ensure that the effect size of all data meets the acceptable statistical power threshold (power>0.8), supporting the reliability of the conclusions drawn.

## Conflict of Interest

The authors declare no conflict of interest

## Author Contributions

R.L., H.X., and Y.S. contributed equally to this work. Conceptualization: R.L., Z.G., Y.W., Data curation/ Investigation: R.L., H.X., X.C., J.L., M.C., C.J., Y.S., X.Y., L.W., L.S., Formal analysis: R.L., H.X., Y.S., X.Y., Resources: C.J., X.D., Z.G., Y.W., Project administration: Z.G., Y.W., X.D., Funding acquisition: Z.G., Y.W., X.D., Methodology/ Validation/ Validation: R.L., Z.G., Y.W., Supervision: Z.G., Y.W., Writing – original draft: R.L., H.X., Y.S., Z.G., Y.W., X.D., Writing – review & editing: R.L., H.X., Y.S., X.D., Z.G., Y.W., X.D.

## Supporting information



Supporting Information

## Data Availability

The data that support the findings of this study are available from the corresponding author upon reasonable request.
