# Supplementary material for: Pharmacological Microglial Inhibition Remodels the Scar Microenvironment to Support Reticulospinal Circuit Reconstruction After Spinal Cord Injury
Source: Adv Sci (Weinh). 2025 Oct 17;13(1):e03966. doi: 10.1002/advs.202503966 (PMC12767085; doi:10.1002/advs.202503966)
Supplement: Supplementary file 1 — Supporting Information [file ADVS-13-e03966-s001.docx]

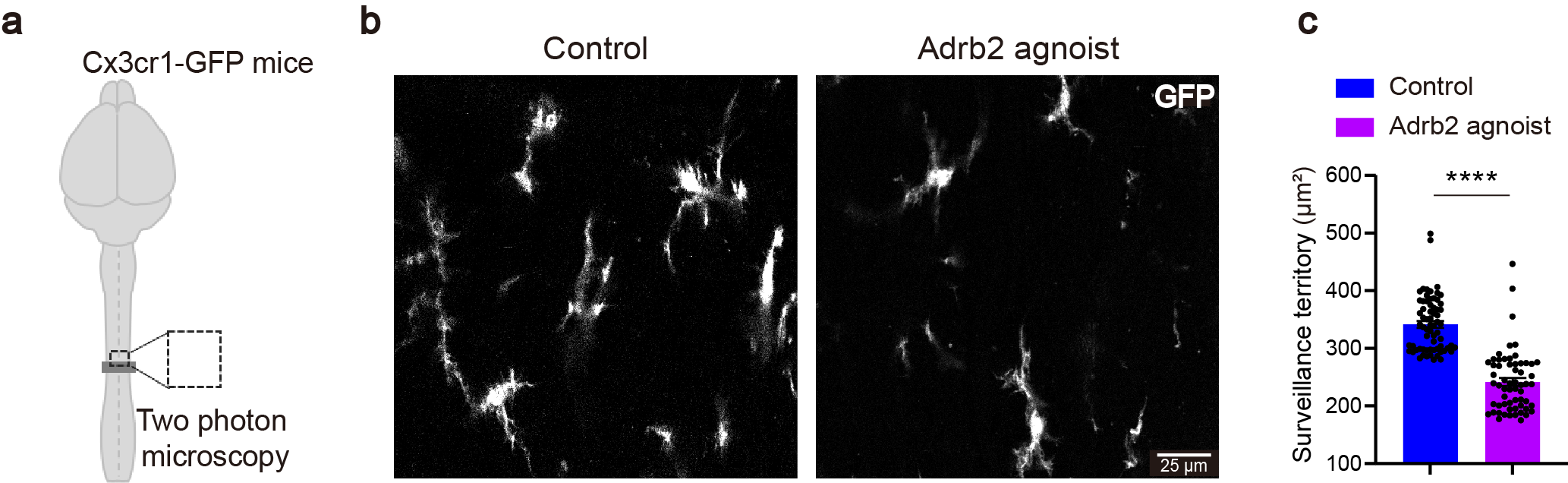


**Extended Data Fig.1| The impact of Adrb2 agonist on microglia in intact spinal cord**

**a,** Schematic diagram of the experimental design. **b,** Representative pictures showing morphological differences between control and treatment group in intact mice. **c,** Quantification of the microglia surveillance territory (cells in each group were detected from 3 mice). **** P < 0.0001. Two-tailed unpaired t-test (c). Data are shown as mean ±s.e.m.


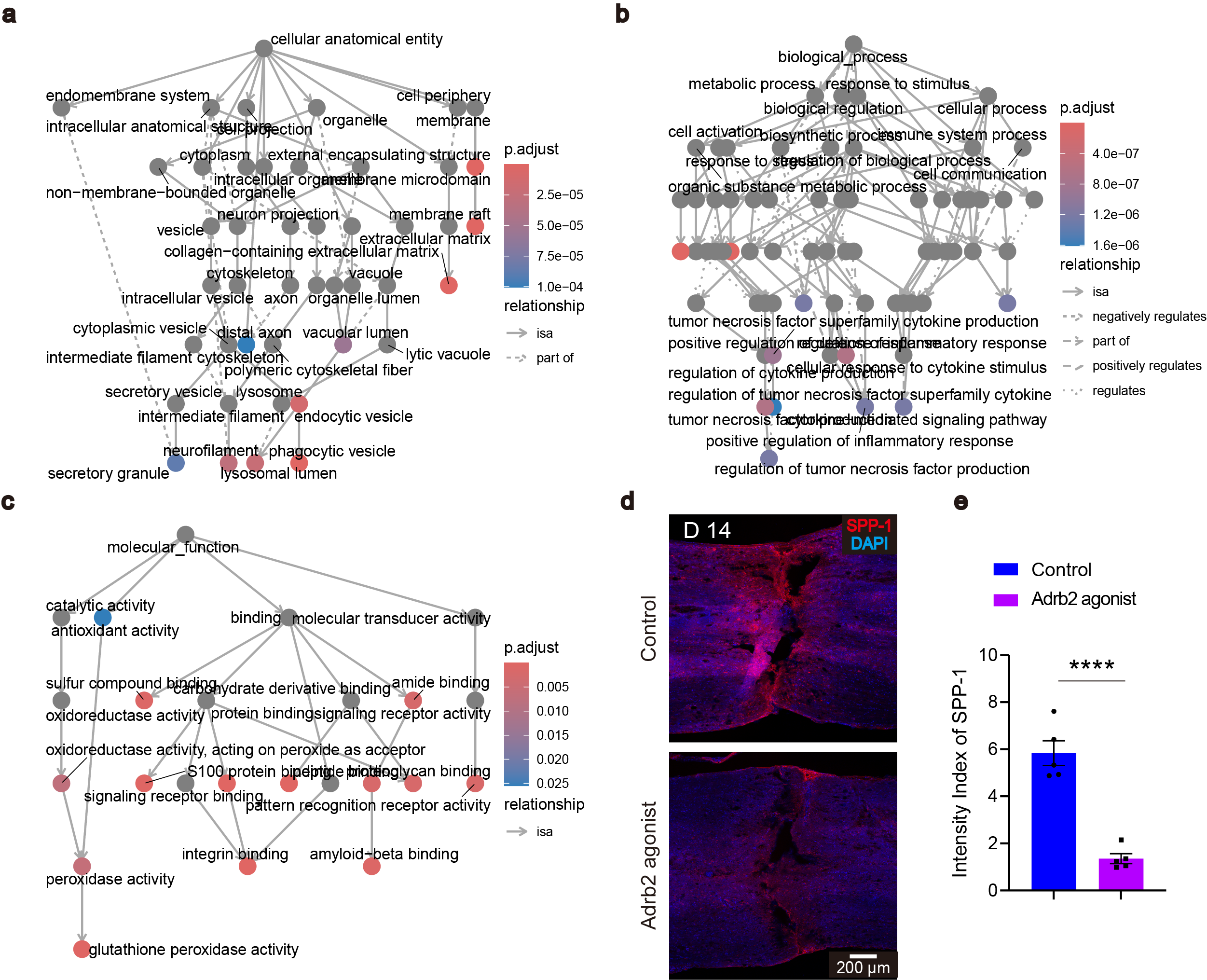


**Extended Data Fig.2| The transcriptomic differences between the injury group and the treatment group**

**a,** Cellular component analysis of the injury group and the treatment group. **b,** Biological process analysis of the injury group and the treatment group. **c,** Molecular function analysis of the injury group and the treatment group. **d,** Representative pictures showing the expression of SPP-1. **e,** Quantification of the SPP-1 immunoreactive intensity (n = 5/5 mice). ** P < 0.01. Two-tailed unpaired t-test (e). Data are shown as mean ±s.e.m.


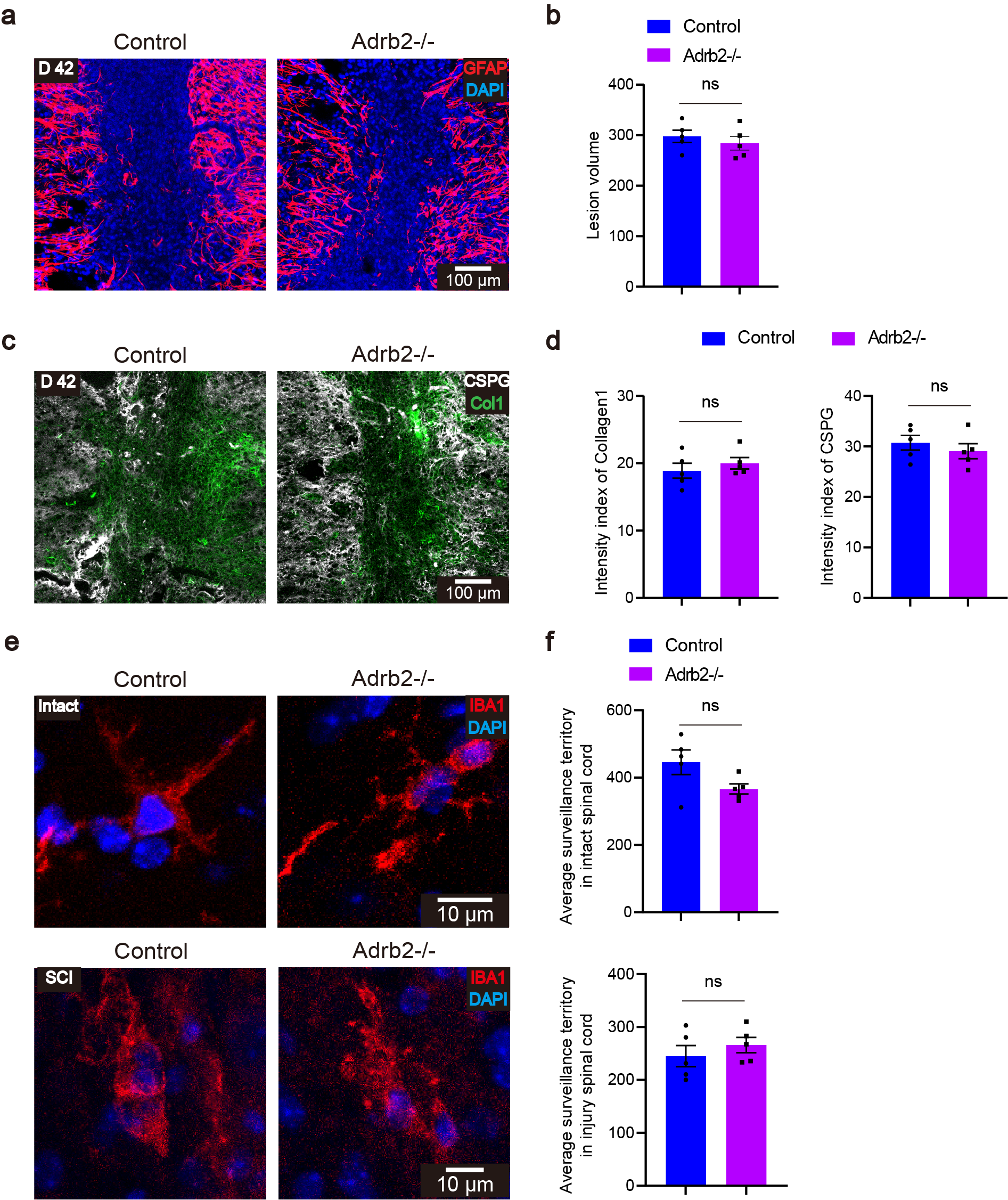


**Extended Data Fig.3| Knockout of Adrb2 does not affect scar formation or microglial morphology**

**a,** Representative sections showing astrocyte scar in different groups at 42 dpi. **b,** Quantification of the lesion volume in different groups (n = 5/5 mice). **c,** Representative sections showing the ECM of the lesion site in different groups at 42 dpi. **d,** Quantification of the indicated immunoreactive intensity in the lesion site (n = 5/5 mice). **e,** Representative sections showing the microglial morphology in different groups. **f,** Quantification of the average surveillance territory (n = 5/5/5/5 mice). ns P > 0.05. Two-tailed unpaired t-test (b, d, f). Data are shown as mean ±s.e.m.


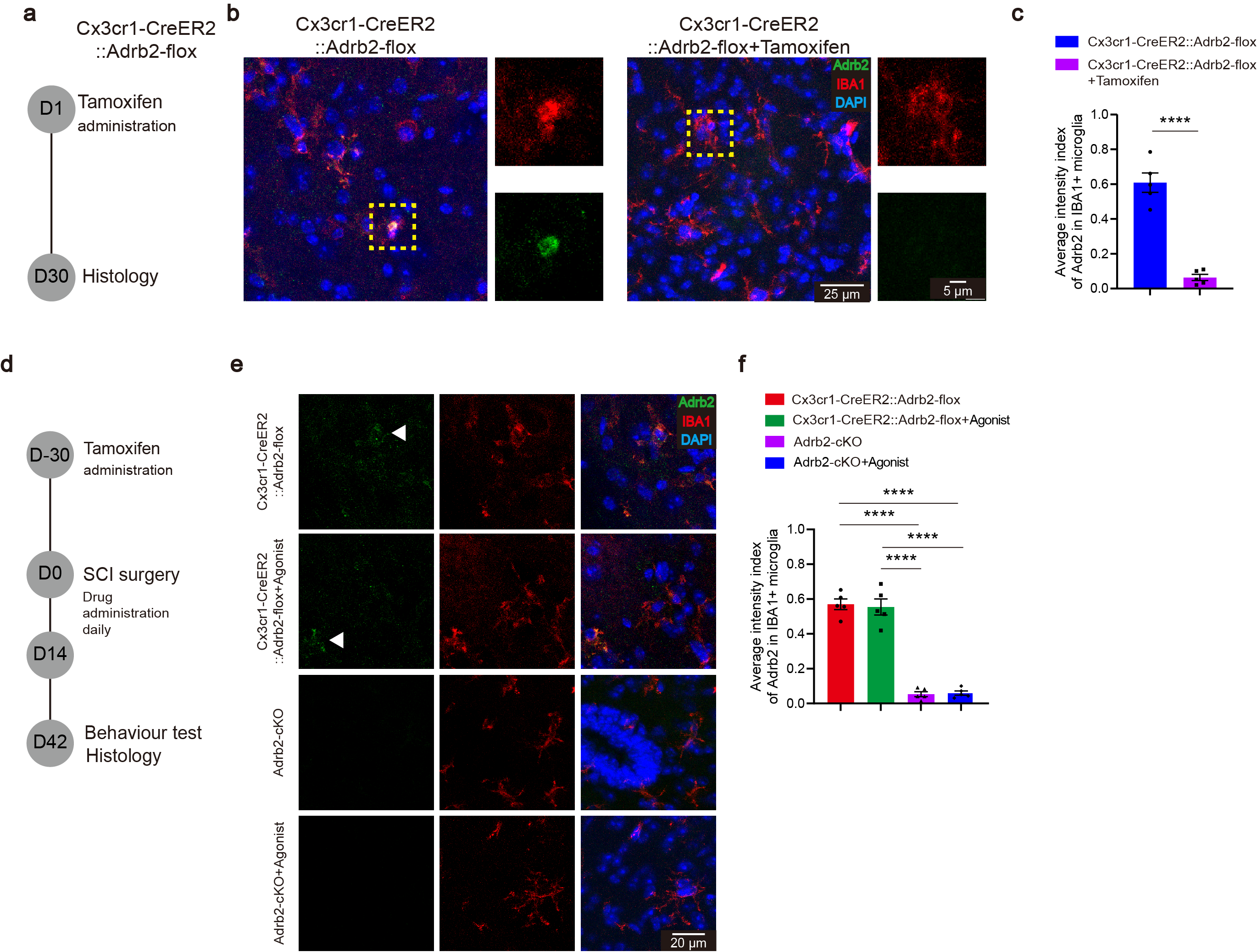


**Extended Data Fig.4| Conditional knockout of Adrb2 in microglia**

**a,** Schematic diagram of the Adrb2 conditional knockout, 100 mg/kg of tamoxifen was administered via intraperitoneal injection for three consecutive days. **b,** Representative pictures showing the expression of Adrb2 at different groups. **c,** Quantification of the expression of Adrb2 in microglia (n = 5/5 mice). **d,** Schematic diagram of the experiment. **e,** Representative images showing the expression of Adrb2 and IBA1 in animals with different treatment. **f,** Quantification of the expression of microglial Adrb2 in different groups (n = 5/5/5/5 mice). *** P < 0.001, **** P < 0.0001. Two-tailed unpaired t-test (c). One-way ANOVA, followed by post hoc Bonferroni correction (f). Data are shown as mean ±s.e.m.


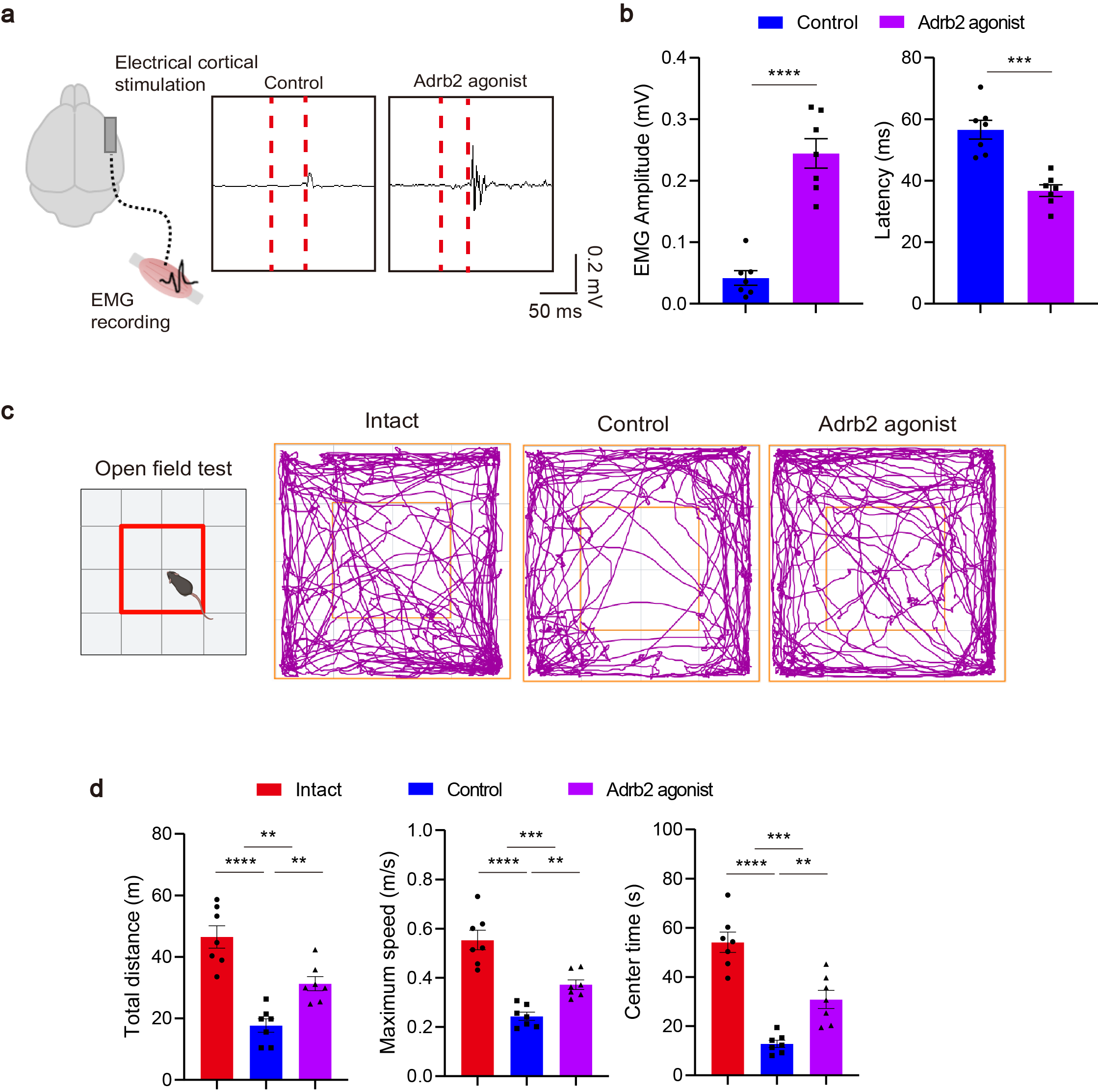


**Extended Data Fig.5| Adrb2 agonist promotes motor recovery**

**a,** Representative EMG responses recorded in TA muscle evoked by cortical stimulations. **b,** Quantification of the amplitude and latency of the EMG signals (n = 7/7 mice). **c**. Trajectory of mice in open field chambers in different groups**. d,** Quantification of the total distance, maximum speed and center times in open field test (n = 7/7/7 mice). ** P < 0.01, *** P < 0.001, **** P < 0.0001. Two-tailed unpaired t-test (b). One-way ANOVA, followed by post hoc Bonferroni correction (d). Data are shown as mean ±s.e.m.


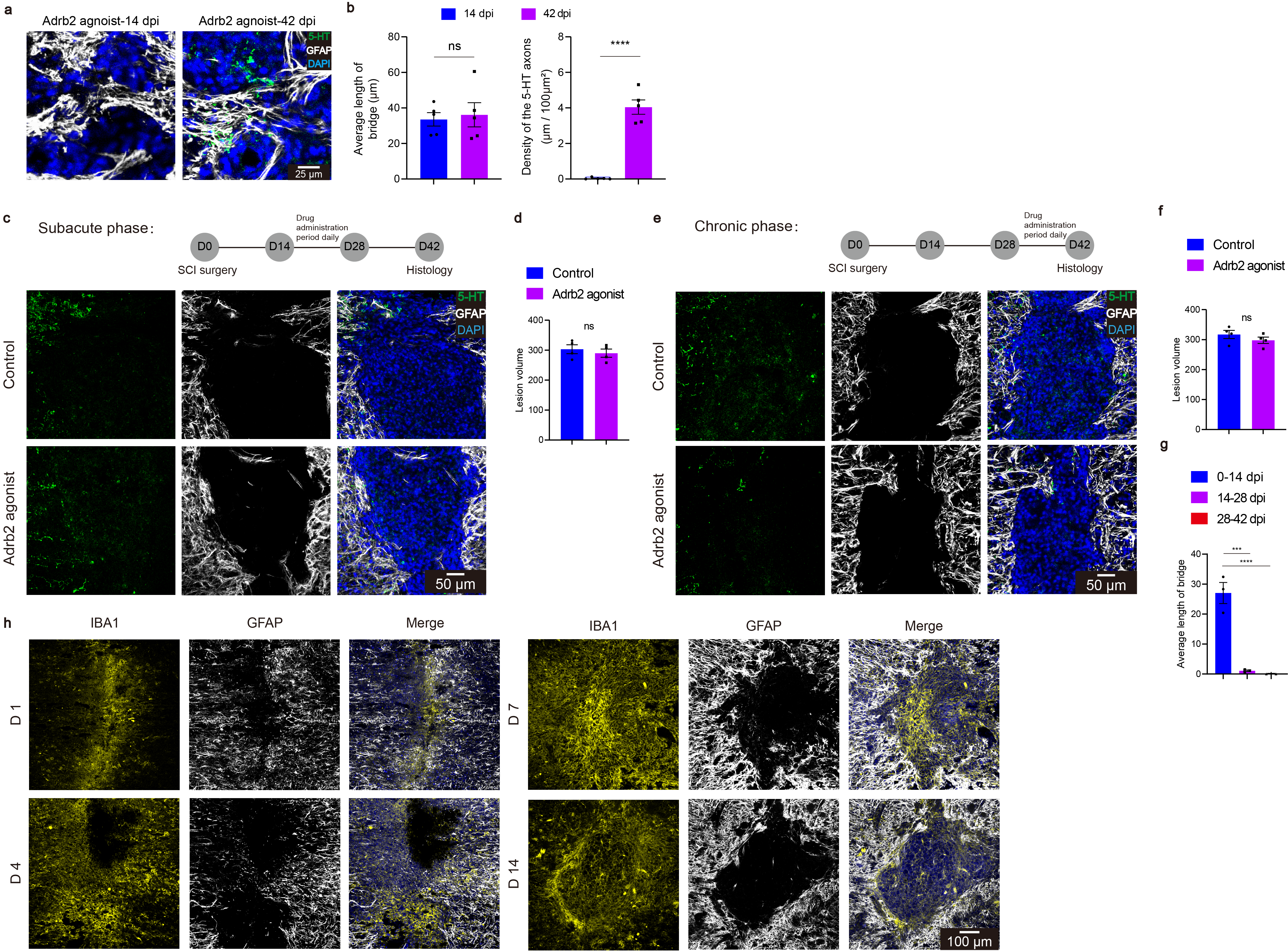


**Extended Data Fig.6| The acute phase is a critical window for microglial inhibition to remodel the scar environment**

**a,** Representative sections showing the astrocyte bridge with 5-HT^+^ axons at different timepoints after treatment. **b,** Quantification of average length of astrocyte bridge (n = 5/5 mice) and quantification of the number of axons crossing the lesion (n = 5/5 mice). **c, e,** Schematic diagram of the experimental design for microglial inhibition therapy and representative sections showing the morphology of scar and 5-HT^+^ fiber in the lesion site at 42 dpi in different groups. **d**, Quantification of the lesion volume in subacute group (n = 4/4 mice). **f**, Quantification of the lesion volume in chronic group (n = 4/4 mice). **g**, Quantification of the average bridge length in different group (n = 3/3/3 mice). **h,** The process of typical scar structure formation. ns P > 0.05, *** P < 0.001, **** P < 0.0001. Two-tailed unpaired t-test (a, d, f). One-way ANOVA, followed by post hoc Bonferroni correction (g). Data are shown as mean ±s.e.m.


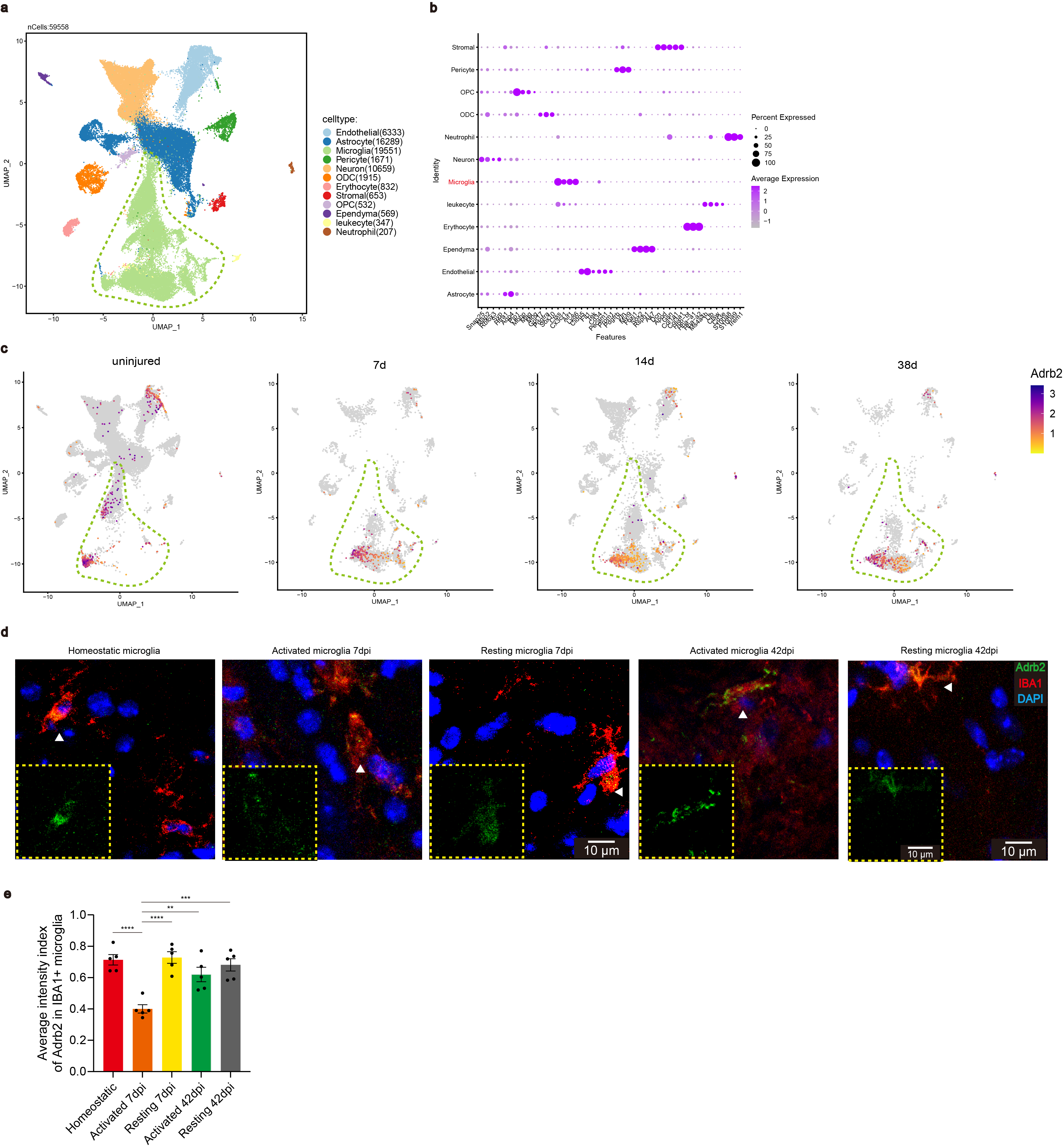


**Extended Data Fig.7| Temporal expression profile of Adrb2 in the SCI environment.**

**a,** UMAP visualization of 59558 spinal cord cells (Li et al., 2022) before and after SCI, including 12 cell types primarily based on feature gene expression, where the green circle indicates microglia.  **b,** Characteristic gene expression maps of 12 cell types. **c,** Expression of Adrb2 at different time points, where the green circle indicates microglia. **d-e,** Representative high magnitude images (d) with quantification (e) showing the expression of Adrb2 at different microglial states and time points after SCI. n= 5 mice at each condition. ns, P > 0.05, ** P < 0.01, *** P < 0.001. One-way ANOVA, followed by post hoc Bonferroni correction (e). Data are shown as mean ±s.e.m.


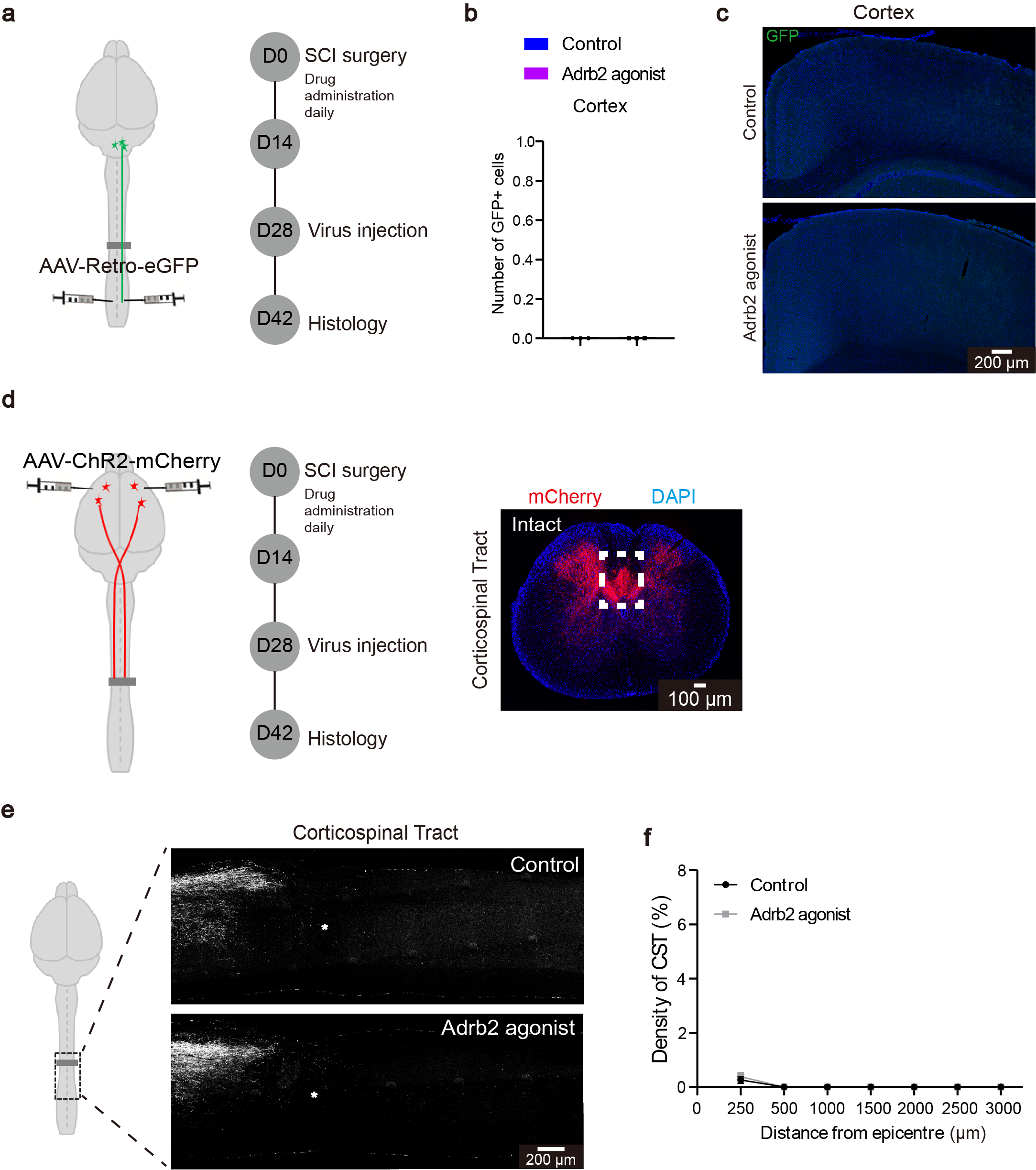


**Extended Data Fig.8| There are differences in the regenerative capacity among descending tracts**

**a.** Schematic diagram of the experimental design for retrograde tracing. **b,** Quantification of the GFP^+^ neurons number in cortex (n = 3/3 mice). **c,** Representative sections showing the GFP^+^ neurons in the motor cortex. **d.** Schematic diagram of the experimental design for CST anterograde tracing with tracing validation in intact spinal cord. **e,** Representative sections showing the projection of CST. **f.** Quantification of the density of CST (n = 3/3 mice). ns P > 0.05. Two-tailed unpaired t-test (b). Two-way ANOVA, followed by post hoc Bonferroni correction (f). Data are shown as mean ±s.e.m.


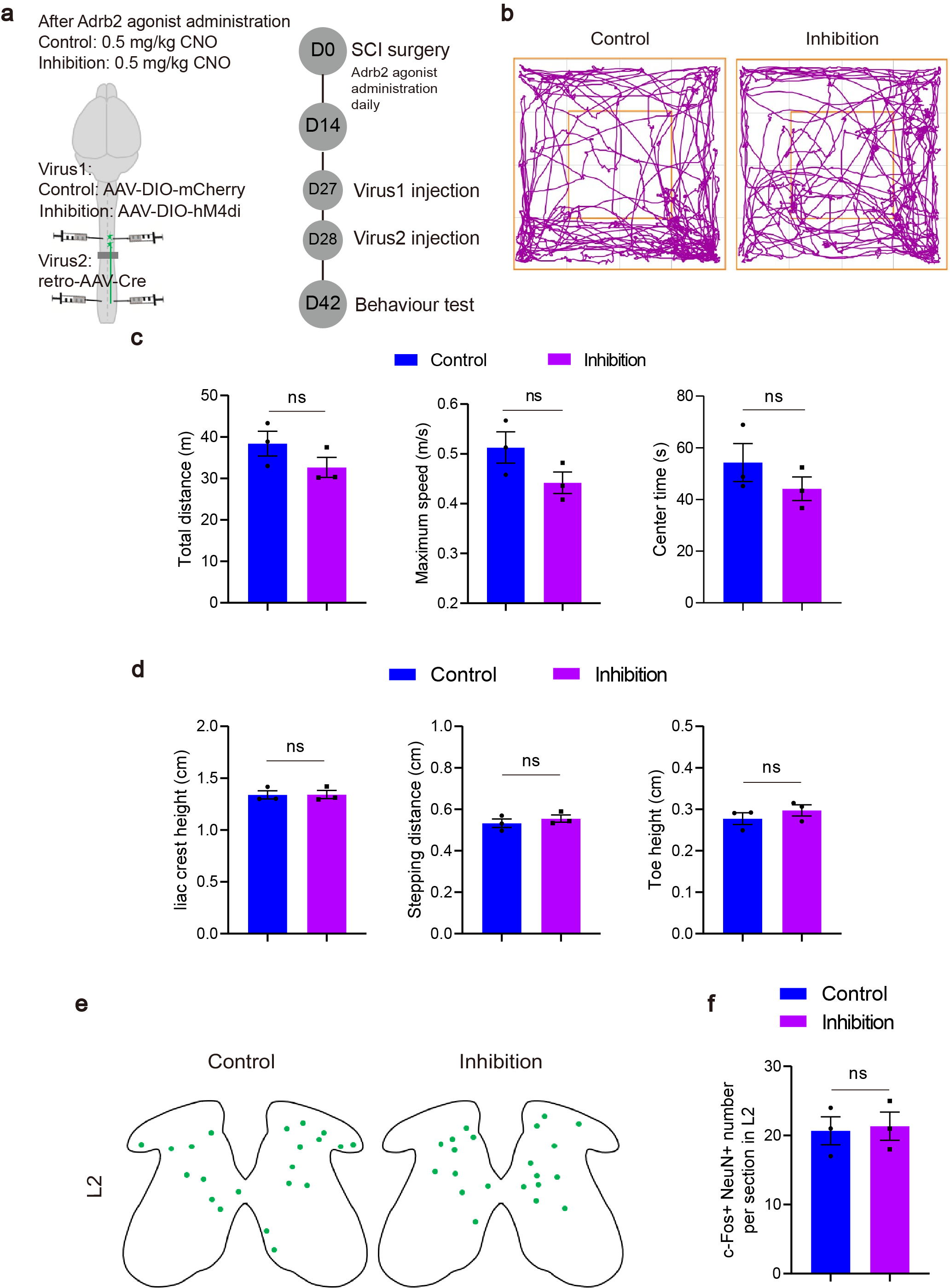


**Extended Data Fig.9| Thoracic propriospinal neurons do not contribute to motor function recovery**

**a,** Schematic diagram of the experimental design for specific inhibition of spared propriospinal neuron. **b**. Trajectory of mice in open field chambers in different groups**. c,** Quantification of the total distance, maximum speed and center times in open field test (n = 3/3 mice). **d,** Quantification of the iliac crest height, stepping distance and toe height (n = 3/3 mice). **e,** Representative sections showing c-Fos^+^ neurons. **f,** Quantification of c-Fos^+^ neurons in L2 sections (n = 3/3 mice). ns P > 0.05. Two-tailed unpaired t-test (c, d, f). Data are shown as mean ±s.e.m.


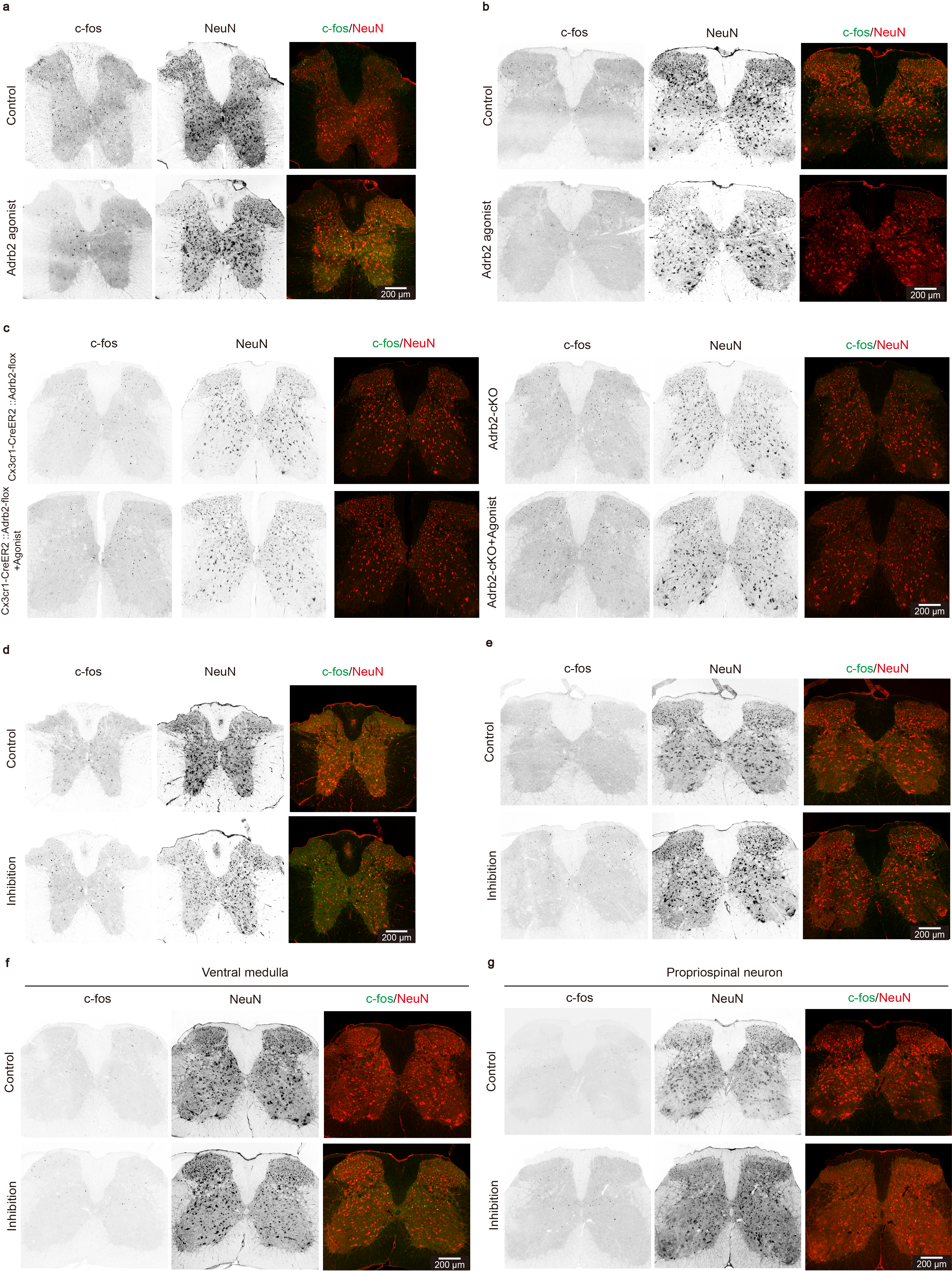


**Extended Data Fig.10| Representative sections showing c-Fos^+^ neurons**

**a, b,** Related to **Fig.3j**

**c,** Related to **Fig.4e**

**d, e,** Related to **Fig.7f**

**f,** Related to **Fig.7l**

**g,** Related to **Extended Data Fig.9e**
